# UHPLC-HRMS analysis combined with feature-based molecular networking methods for systematic identification of chemicals in AnShenDingZhiLing and its absorbed metabolites

**DOI:** 10.3389/fchem.2025.1647159

**Published:** 2025-09-03

**Authors:** Tai Han, Deqing Ni, Wanqing Zhu, Dan Fang, An Kang, Yumiao Wu, Jichao Sun

**Affiliations:** 1 Department of Science and Technology, Guangxi University of Chinese Medicine, Nanning, China; 2 School of Pharmacy, Nanjing University of Chinese Medicine, Nanjing, China; 3 Department of Pediatrics, The First Affiliated Hospital of Guangxi University of Chinese Medicine, Nanning, China

**Keywords:** UHPLC-HRMS, feature-based molecular networking, flavonoids, integrated strategies, prototype and metabolite components

## Abstract

**Introduction:**

AnShenDingZhiLing is an effective Chinese herbal formula that is clinically used to treat pediatric attention deficit hyperactivity disorder (ADHD). In terms of the overall prescription, it has shown great efficacy in alleviating ADHD symptoms and holds broad clinical application prospects. However, due to the lack of *in vitro* and *in vivo* studies, the chemical components and metabolites of AnShenDingZhiLing remain poorly understood, which also hinders research into the pathogenesis of ADHD.

**Methods:**

In this study, we established a rapid and efficient method employing the UHPLC-HRMS system and integrated multiple strategies to systematically characterize and identify the chemical profiles and drug metabolites in the biological samples of AnShenDingZhiLing and AnShenDingZhiLing-treated rats.

**Results and discussion:**

243 compounds (including 60 flavonoids, 50 terpenoids, 24 phenylpropanoids, 18 alkaloids, 18 anthraquinones, 16 phenylethanoid glycosides, 13 phenolic acids, nine xanthones, nine oligosaccharides, eight phthaleins, eight naphthopyrones, four organic acids, four aromatic aldehydes, and two diarylheptanoids) were characterized. Following the administration of AnShenDingZhiLing to rats, a total of 110 compounds related to Chinese herbal medicine ingredients were identified in the plasma and cerebrum samples. The primary metabolic pathways of chemicals derived from AnShenDingZhiLing can be summarized in methylation, demethylation, hydrolysis, hydroxylation, sulfation, and glucuronidation. In summary, through this rapid and accurate analytical method, the comprehensive chemical profiles of AnShenDingZhiLing and its metabolites were characterized. Additionally, in this study, we provide essential analytical thinking and scientific evidence for exploring the material basis of AnShenDingZhiLing efficacy further.

## 1 Introduction

The incidence of attention deficit hyperactivity disorder (ADHD) is progressively higher among children and adolescents, and it still has the possibility of affecting the patient’s normal life in adulthood (Li et al., 2024). Pharmacological treatments including methylphenidate and atomoxetine and non-pharmacological treatments including parent training, behavioral therapy, and dietary modifications have been shown to be partially effective. Traditional Chinese medicine (TCM) shows unique therapeutic benefits in treating numerous diseases. Recently, research workers mainly focused on TCM due to the significant therapeutic efficacy on neurological diseases (Moreira et al., 2023). However, the corresponding targets and efficacy of the TCM ingredients are not very clear due to the diversity of chemical structures and complex material basis. AnShenDingZhiLing (ASDZL) comprises 12 herbal medicines, including *Scutellaria baicalensis* Georgi (Scu), *Bupleurum chinense* DC. (Bup), *Angelica sinensis* (Oliv.) Diels (Ang), *Rehmannia glutinosa* (Gaertn.) Libosch. ex DC. (Reh), *Uncaria rhynchophylla* (Miq.) Miq. ex Havil. (Unc), *Senna obtusifolia* (L.) H.S. Irwin and Barneby (Sen), *Forsythia suspensa* (Thunb.) Vahl (For), *Acorus calamus* var. angustatus Besser (Aco), *Alpinia oxyphylla* Miq. (Alp), *Curcuma aromatica* Salisb. (Cur), *Polygala tenuifolia* Willd. (Pol), and *Bambusa textilis* McClure (Bam). ASDZL has the efficacy of clearing the heart and calming the liver, eliminating phlegm and resuscitation, and soothing and calming the mind (Yaqun et al., 2022). Catalpol, a main constitute in ASDZL, can inhibit neuronal apoptosis, promotes myelination, and enhances brain-derived neurotrophic factor (BDNF) expression by modulating crucial proteins involved in prefrontal cortical maturation. Baicalin derived from Scu ameliorates ADHD symptoms by significantly increasing dopamine levels in the striatum. Saikosaponin A derived from Bup downregulates dopamine transporter levels and increases BDNF expression in the brain. Although there have been correlational studies on catalpol, baicalin, and saikosaponin A (Jichao et al., 2017; Yuan et al., 2019; Zhou et al., 2019), for ASDZL, the intricate material basis is still poorly understood, and the mechanism of action is yet to be clarified. Hence, there is an urgent need to elucidate the overall variety of the chemical profiles of ASDZL (Su et al., 2023).

For TCM, detailed analysis of its chemical composition facilitates the discovery, isolation, and purification of bioactive components. Currently, the development of more diverse analytical techniques has provided innovative methods to elucidate its material basis. Ultra-high-performance liquid chromatography–high-resolution mass spectrometry (UHPLC-HRMS) has been broadly used to characterize the complex components of TCM due to high-efficiency chromatographic separation, high-sensitive mass spectrometry performance, and a large amount of mass spectrum information (Hong et al., 2025). Nevertheless, research workers expended a lot of time and energy to analyze a large number of UHPLC-HRMS raw data. The Global Natural Products Social Molecular Networking (GNPS), an open-access platform, facilitates mass spectrometry data analysis and visual annotation of compounds, which is a huge advantage for discovering potential natural products with biological activity. Feature-based molecular networking (FBMN) utilizes the GNPS platform to provide various and reachable applications in comparison with expensive commercial mass spectrometry databases (Wang et al., 2016; Nothias et al., 2020). FBMN integrates abundance mass spectrometry data, alignment tools, and chromatographic behavioral features of natural products (Katajamaa et al., 2006; Pluskal et al., 2010). FBMN can generate molecular networks (MNs) using the GNPS platform. The MN clusters structurally related compounds based on similar spectral information, and these compounds tend to have similar backbones and fragmentation pathways (Chen et al., 2024). GNPS is a powerful tool to discover potential novel natural products and distinguish isomers for TCM (Pakkir Shah et al., 2024).

In this study, the UHPLC-HRMS method was employed to obtain dependable mass spectrometry data of ASDZL. We proposed the integrated strategy that organically combines diagnostic ion filtering (DIF), neutral loss filtering (NLF), and MN strategies (Zhu et al., 2023). First, the DIF, NLF, NRF, and mass spectrometric fragmentation pathways of different compound types were systematically summarized using reference standards and literature reports. In addition, unknown compounds in ASDZL were inferred using FBMN analysis on the online workflow GNPS. A detailed non-targeted chemical analysis of ASDZL was performed by integrating the characteristic structural analysis of the compounds with the MN analysis (Han et al., 2025). Finally, with the reliability and accuracy of the above work being ensured, we focused on the prototype components of ASDZL present in the blood and brain. *In vivo* metabolic components were extrapolated and predicted using MetabolitePilot™ software and literature reports. This experiment systematically characterized the material chemical basis of ASDZL and utilized the visualization function of MN. The findings not only establish a scientific foundation for subsequent ASDZL studies but also propose a novel analytical approach for processing TCM mass spectrometry data.

## 2 Materials and methods

### 2.1 Experimental materials

Methanol (MeOH) (HPLC grade) and acetonitrile (ACN) (LC–MS grade) were purchased from Merck Co., Ltd. (Darmstadt, Germany), and formic acid (FA) (LC–MS grade) was obtained from Thermo Fisher Technology Co., Ltd. Ultrapure water purification was carried out using a Milli-Q system (Millipore, Bedford, MA, United States of America). Detailed information on the herbal medicines and reference standards used in this study can be found in the Supplementary Material.

### 2.2 Preparation of the ASDZL formula

Twelve herbal medicines (Scu, Bup, Ang, Reh, Unc, Sen, For, Aco, Alp, Cur, Pol, and Bam) in the ASDZL formula were mixed in a weight ratio of 5:3:5:5:5:5:5:5:5:3:5:3, and then the herbal mixture was soaked in an 8-fold volume of distilled water for 1 h. The herbal mixture was subsequently decocted twice with distilled water (1 h per extraction). Finally, the solution was collected and concentrated to 0.89 g/mL using a rotary evaporator.

### 2.3 Animal experiments

Twelve male Sprague–Dawley (SD) rats (180–220 g, SPF grade) were offered by Jiangsu Qinglongshan Biotechnology Co. Ltd. (license no. SCXK-Zhe-20,230,077). The animal experiment protocol was approved by the Animal Ethical Committee of the Guangxi University of Chinese Medicine, and all animal treatments were implemented in agreement with the Guide for the Care and Use of Laboratory Animals of the US National Institutes of Health. The rats were kept standardly in a specified pathogen-free condition (indoor temperature, 23°C ± 3°C; relative humidity, 40%–60%; 12 h light/dark cycle). All rats were allowed free access to food and water *ad libitum* for 7 days. Animals were randomly allocated to two groups: the blank group (n = 6) and the ASDZL group (n = 6). The rats in the ASDZL group were administered ASDZL (uniformly at 9:00 a.m. and 15:00 p.m.) at 20.16 g/kg, whereas rats in the blank group were administered the same volume of distilled water twice daily. Finally, plasma and cerebrum samples were collected following the final oral administration of ASDZL extract.

### 2.4 Preparation of ASDZL extract

An amount of 200 µL of ASDZL (0.89 g/mL) solution was accurately pipetted to each 2-mL EP tubes, and then 600 µL of methanol was added. After mixing, the solution was ultrasonically extracted for 30 min. The supernatant was collected after centrifugation at 12,000 rpm for 10 min at 4°C. The vacuum centrifugal concentrator (Thermo Fisher Scientific) was employed to evaporate the supernatant, which was transferred to a new tube. The residues were redissolved with 300 µL of methanol. The solvent was vortexed for 3 min and centrifuged at 12,000 rpm for 10 min at 4°C. Finally, 50 µL of the supernatant was accurately aspirated, and 200 µL of methanol was added. The solution was aspirated in the injection bottle and prepared for UHPLC-HRMS analysis.

### 2.5 Biological sample preparation

Rat blood samples were acquired in the heparinized centrifuge tubes (1.5 mL) at 1 h and 4 h after the last ASDZL extract administration. Rat plasma was obtained by centrifuging at 4,000 rpm for 10 min at 4°C. An amount of 600 µL acetonitrile was added to 200 μL of plasma samples to precipitate proteins. Next, the supernatant was obtained by centrifuging at 12,000 rpm for 10 min at 4 °C. The appropriate amount of the supernatant was evaporated, and then 300 µL methanol was added to redissolve the residues. Finally, the supernatant was aspirated in the injection bottle and prepared for the analysis after centrifugation.

Similarly, rat cerebrum samples were collected. A total of 300 mg of cerebrum samples was added to each 2-mL EP tubes. Subsequently, 600 µL of physiological saline was added. The mixture was homogenized at 60 Hz for 5 min at 4°C using two zirconia beads. After centrifugation at 12,000 rpm for 10 min at 4°C, 300 µL of the brain tissue homogenate was aspirated and mixed with 300 µL acetonitrile to precipitate proteins. The appropriate amount of the supernatant was evaporated, and then the residues were redissolved in 200 µL methanol. Finally, the supernatant was aspirated in the injection bottle and prepared for the analysis after centrifugation.

### 2.6 UHPLC-HRMS analysis

#### 2.6.1 UHPLC conditions

Liquid chromatography separation was performed on an ExionLC™ 2.0 UHPLC system (AB SCIEX). The column Accucore™ C_18_ (2.1 × 100 mm, 2.6 µm, Thermo Fisher Scientific™) was employed for the separation of various constituents at 40°C. The mobile phases consisted of water containing 0.1% formic acid (A) and acetonitrile (B). The UHPLC elution procedure was implemented in accordance with the following gradient: 0–2 min, 10% B; 2–28 min, 10%–80% B; 28–30 min, 80% B; 30–30.1 min, 80%–10% B; 30.1–32 min, 10% B. The flow rate was set at 0.4 mL/min. The injection volume was 4 μL.

#### 2.6.2 MS conditions

Mass spectrometric analysis was carried out on the ZenoTOF™ 7600 QTOF mass spectrometer (AB SCIEX, United States of America). Comprehensive mass spectrometric data of the constituents were acquired using the information-dependent acquisition (IDA) mode. The MS^1^ mass range was set at *m/z* 100–1,250 and the MS^2^ mass range was set at *m/z* 80–1,250 both in the positive and negative ion modes. Dynamic background subtraction (DBS) was turned on to reduce interference. Other parameters were set as follows: curtain gas (CUR), 35 psi; atomizing gas (GS1), 55 psi; auxiliary gas (GS2), 55 psi; accumulation time, 0.05 s; the ESI temperature, 550°C; the ion spray voltage was 5,500 V in the positive ion mode and −5,500 V in the negative ion mode; the collision energy (CE) was 35 V in the positive ion mode and −35 V in the negative ion mode; the de-clustering potential (DP) was 80 V in the positive ion mode and −80 V in the negative ion mode.

### 2.7 FBMN annotation on GNPS

The GNPS platform provides FBMN for advanced MS data analysis (https://gnps.ucsd.edu). Utilizing the MSConvertGUI software version 3.0, which was from the open-source, cross-platform ProteoWizard (https://proteowizard.sourceforge.io/), the raw data from UHPLC-HRMS were converted to “mzML” format files. The “peak picking” was selected from different filters. Under the vendor algorithm, MS level 1–2 was added. Furthermore, the MS data were imported to MZmine software 4.3.0 (https://github.com/mzmine/mzmine/releases/tag/v4.3.0). For MS data, the mass detection thresholds were set to a noise level of 1,000 for MS^1^ and 100 for MS^2^. Chromatogram builder was performed with the following parameters: MS level filter (level = 1); minimum consecutive scans, five; minimum absolute height, 7,000; minimum intensity for consecutive scans, 3,000; *m/z* tolerance, 0.005 Da or 10.0 ppm. The local minimum feature resolver was implemented with the chromatographic threshold of 90.0%, minimum absolute height of 7,000, peak duration range of 0–1 min, and five minimum scans. The main ^13^C isotope filter parameters were as follows: *m/z* tolerance, 0.001 Da or 5.0 ppm; retention time tolerance, 0.01 min; maximum charge of one and monotonic shape check. The join aligner was implemented with *m/z* of 0.001 Da or 5.0 ppm, weight for *m/z* of 3, retention time tolerance of 0.1 min, and weight for retention time of one. Then, the feature list rows filter was performed with feature with MS^2^ scan and the never remove feature with MS^2^. Finally, the processed feature list was uploaded onto the GNPS online platform through WinSCP software version 6.3.5 (https://winscp.net/eng/download.php). The FBMN parameters were set as follows: precursor ion mass tolerance of 0.02 Da, fragment ion mass tolerance of 0.02 Da, minimum pairs cosine of 0.70, minimum matched fragment ions of six, and library search min matched peaks of six. The data of the FBMN were analyzed by Cytoscape software version 3.10.2 (https://www.cytoscape.org/).

## 3 Results and discussion

### 3.1 Summary of integration strategy

Given that ASDZL consists of twelve TCMs, identifying its numerous chemical compounds is particularly challenging. Therefore, we developed a fast, effective, and accurate integrated strategy for comprehensive characterization of ASDZL’s chemical constituents. The procedure primarily comprises the following steps: (1) for HRMS ZenoTOF™ 7600, Zeno™ Trap on, the IDA technology mode and DBS can more sensitively obtain accurate MS information. ASDZL data were acquired by using UHPLC-HRMS. Then, the chemical components’ database of ASDZL was established in conjunction with literature reports as well as the TCMSP database, including precise molecular mass, compound names, and major fragment ions. (2) The preprocessed MS data were implemented by FBMN assignment at the online platform, GNPS. The annotated results of MN were carefully analyzed in conjunction with reference standards and literature reports. (3) Key MS information on DIF, NLF, and the fragmentation pathway were summarized for these major types of components. Annotation information of potential components from MN and the reference standards in ASDZL were integrated to characterize the chemical profiles of ASDZL. Subsequently, chromatographic data and MS data were analyzed using PeakView™ software (AB SCIEX). (4) With the reliability and accuracy of the above workflow established, this step focused on identifying the prototypical components absorbed into biological samples. The DIF and NLF strategies were integrated to extrapolate and predict *in vivo* metabolic components using MetabolitePilot™ software and literature reports. Finally, the metabolic pathways of the predominant chemical constituents in ASDZL were systematically summarized.

### 3.2 Annotation of components by feature-based molecular networking

**FIGURE 1:**
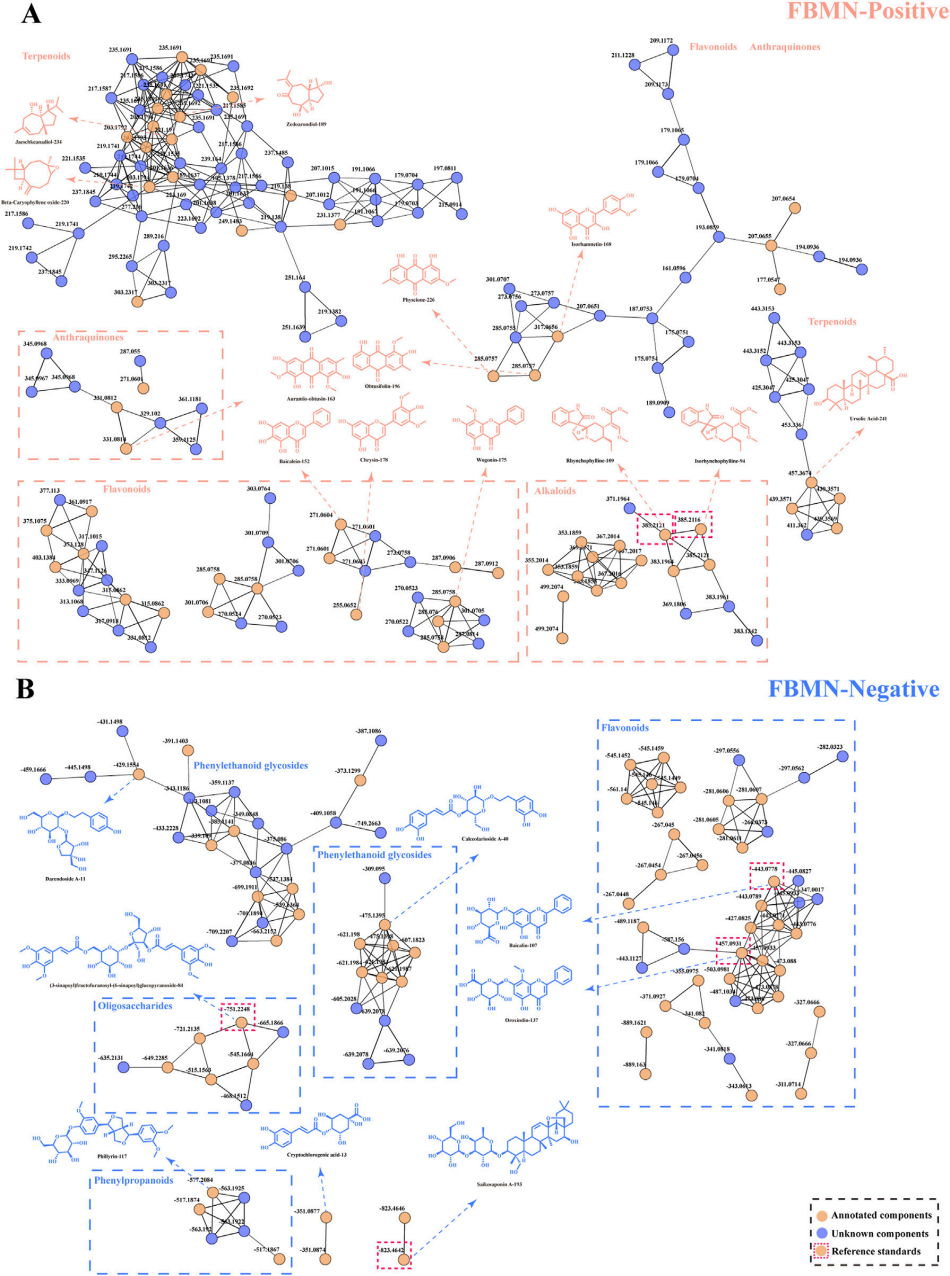
Visual analysis of FBMN in the positive **(A)** and negative **(B)** ion modes. Blue nodes represent unknown compounds, and orange nodes represent annotated compounds. Nodes with red borders were identified as reference standards.

The number of components in ASDZL is so large that it is not sufficient to characterize the full chemical profile by literature reports. Hence, the data were processed using MZmine to generate MN through the online platform FBMN, and the MN provided more comprehensive MS information to complement and modify the chemical profile of ASDZL (Heuckeroth et al., 2024). Positive (Supplementary Figure S1) and negative ion mode (Supplementary Figure S2) MN were generated by FBMN. Blue nodes represent unknown compounds, and orange nodes represent annotated compounds. The positive ion mode comprised a total of 610 nodes, 489 clustered nodes, and 121 non-clustered nodes. Among these, 141 molecular nodes were annotated through MS^2^ spectral comparison with reference spectra in the GNPS database. The negative ion mode comprised a total of 244 nodes, 139 clustered nodes, and 105 non-clustered nodes, and 77 nodes were annotated through MS^2^ spectral comparison with reference spectra in the GNPS database. In terms of the clustered nodes, 29 of 64 positive FBMN clusters (nodes≥ 2) and 16 of 28 negative FBMN clusters (nodes≥ 2) contained annotated compounds (Li et al., 2023; Qu et al., 2023). With respect to the non-clustered nodes, 14 nodes of positive FBMN and eight nodes of negative FBMN were annotated by the GNPS database. The MS^2^ fragments and structure type of the annotated nodes are relatively similar in analyzing the clusters that were primarily annotated. Thus, components in the same cluster are likely to have the same diagnostic ions, neutral losses, and fragmentation pathways. Flavonoids, phenylethanoid glycosides, and triterpenoids were annotated in both the positive and negative FBMN clusters, and nodes with red borders were identified as reference standards (Figure 1). Alkaloids and anthraquinones were only annotated in positive FBMN, and organic acids and oligosaccharides were only annotated in negative FBMN. A sufficient number of nodes that share similar MS^2^ spectral features with the annotated nodes and may be potentially new compounds have still not been annotated. Particularly, in the example of alkaloids isomers, the annotations of isorhynchophylline (compound 94) and rhynchophylline (compound 109) were consistent with those of the reference standards and the literature reports, which suggests that there is a great potential to identify isomers by FBMN workflow. Indeed, the rapid advancement of the UHPLC-HRMS technology coupled with the FBMN strategy has greatly enhanced the scientific analysis of TCMs. As an irreplaceable analytical instrument, UHPLC-HRMS poses challenges due to its large data volume and the inference of isomers. Therefore, we rely on the FBMN strategy to quickly annotate compounds and isomers. However, the FBMN analysis is limited by the GNPS database, resulting in false positives and unannotated compounds (Zhang et al., 2024a). After comparing the other reference standards and screening out the unreasonable redundant nodes and wrong annotations by DIF and NLF strategies, 61 components were finally obtained from the MN. A total of 61 compounds included 21 flavonoids, 11 alkaloids, eight terpenoids, six phenylpropanoids, four anthraquinones, six phenylethanoid glycosides, three oligosaccharides, one phenolic acid, and one organic acid (Supplementary Table S1).

### 3.3 Utilization of DPI and NLF strategies of main structure types

Multiple structure types were identified in ASDZL. Diagnostic ions and neutral losses of flavonoids, terpenoids, anthraquinones, alkaloids, phenylethanoid glycosides, oligosaccharides, xanthones, and phenolic acids were summarized based on the MS information of 11 reference standards and two components from the MN in the positive and negative ion modes (Supplementary Table S2). Diagnostic ions are mainly obtained for retro-Diels–Alder (RDA) cleavage, losses of glycosides, and specialized skeletons. Neutral molecules were mostly glycosides, H_2_O, and CO (van Dinteren et al., 2021).

### 3.4 Analysis of components from ASDZL

**FIGURE 2:**
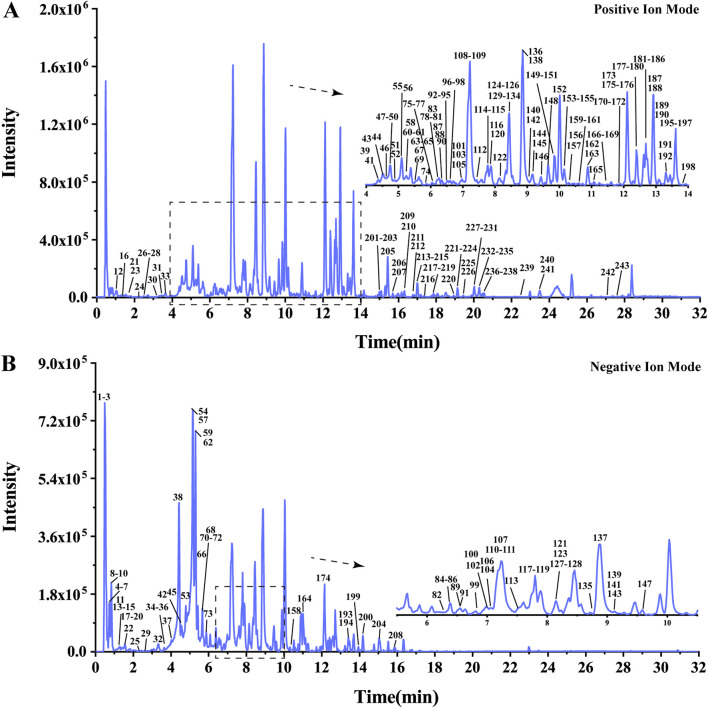
Base peak chromatogram (BPC) of ASDZL in UHPLC-HRMS positive **(A)** and negative **(B)** ion modes.

**FIGURE 3:**
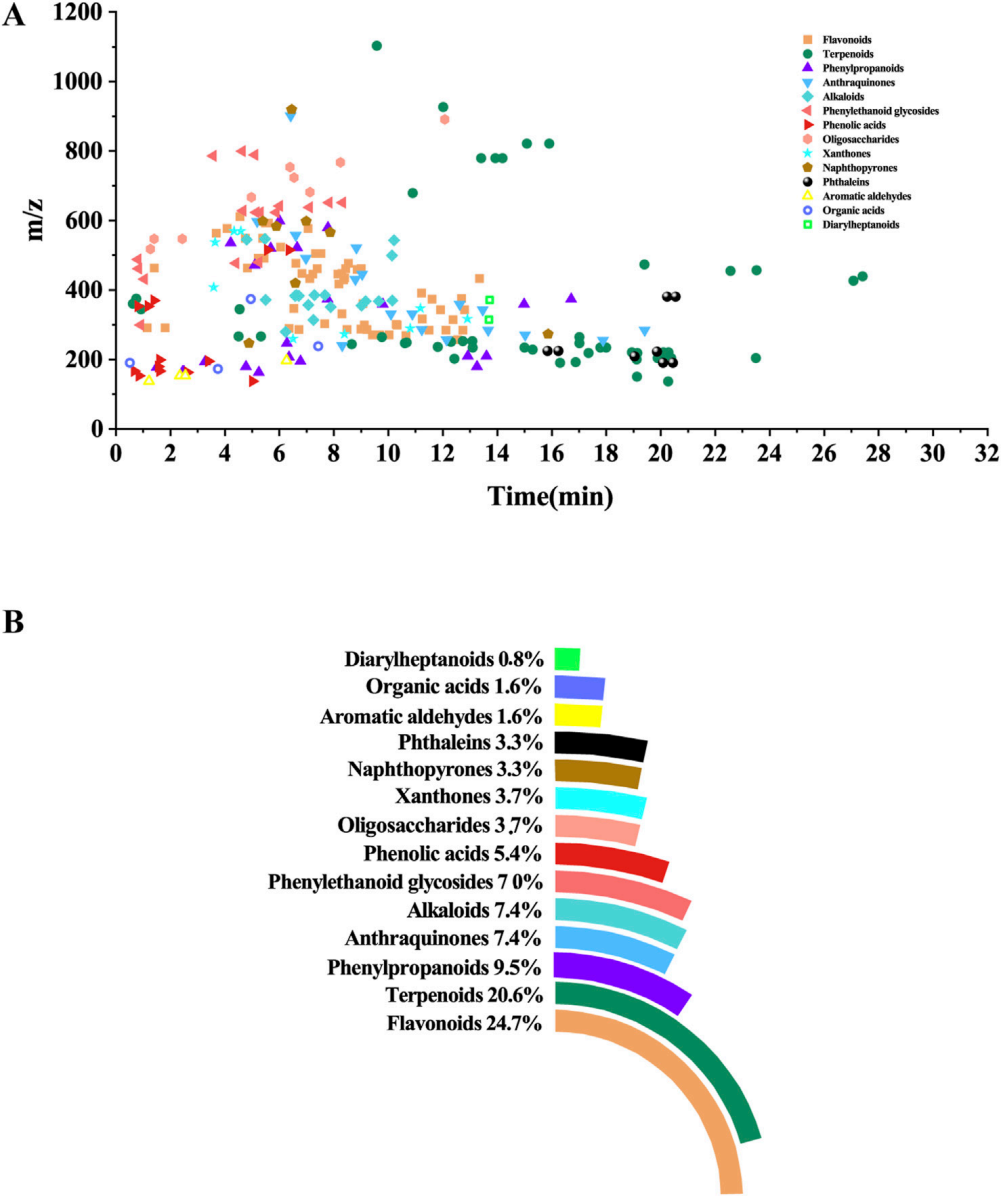
Distribution and chemical structure of 243 components characterized from ASDZL. **(A)** Distribution of retention time, accurate mass, and chemical structure in the scatterplot. **(B)** Proportion of 14 chemical structures from donut and pie chart.

**FIGURE 4:**
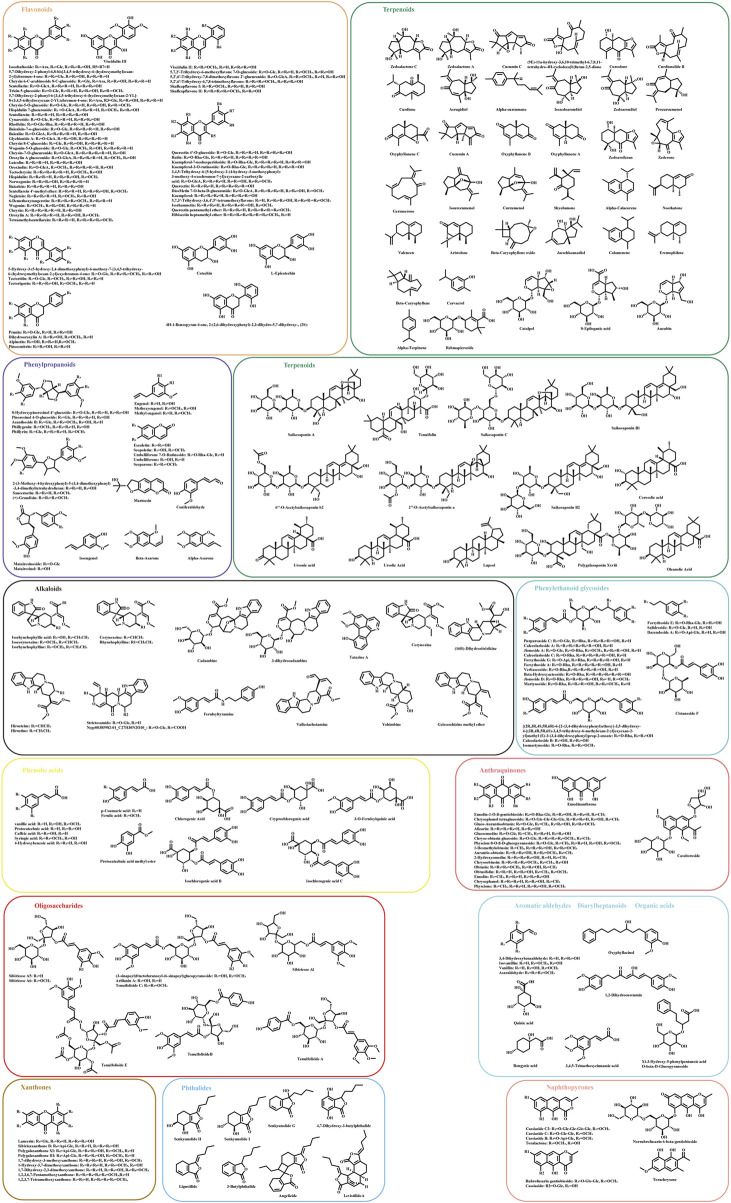
Chemical structural formulas of ASDZL are classified in different types.

The UHPLC-HRMS base peak chromatograms (BPCs) of ASDZL could be seen in Figure 2. The BPCs of the reference standards were exhibited in Supplementary Figure S3. The adduct ions were set as follows: positive ion mode: [M + H]^+^, [M + NH_4_]^+^, and [M + H-H_2_O]^+^; negative ion mode: [M–H]^-^ and [M + HCOO]^-^. Chemical constituents of ASDZL and their detailed characteristics are shown in Supplementary Table S3. A total of 243 compounds (including 60 flavonoids, 50 terpenoids, 24 phenylpropanoids, 18 alkaloids, 18 anthraquinones, 16 phenylethanoid glycosides, 13 phenolic acids, nine xanthones, nine oligosaccharides, eight phthaleins, eight naphthopyrones, four organic acids, four aromatic aldehydes, and two diarylheptanoids) were characterized from ASDZL. Among these, 61 compounds were annotated by GNPS database matching, whereas 17 compounds were identified using authentic reference standards. The distribution of these compounds and the types of structure are intuitively illustrated in Figure 3. Organic acids and the components with glycosides exhibit higher polarity and are usually ahead in retention time. However, flavonoids, alkaloids, and triterpenoids have relatively late retention times. In terms of numbers, the main chemical structure types of ASDZL are flavonoids, terpenoids, and phenylpropanoids. Jingning fang is used for the treatment of ADHD. Both Jingning fang and ASDZL contain the same three herbal medicines, namely, Reh, Pol, and Aco. The main compounds of Jingning fang are triterpenoids, oligosaccharides, and phenolic acids, which are also partially present in ASDZL (Yang et al., 2017). All the chemical structures of the constituents in ASDZL are classified in Figure 4. The mass error of 242 compounds was within five, and only compound 55 reached −5.844, but it was confirmed to be 4-hydroxybenzoic acid by accurate MS^2^ information. Therefore, summarizing the fragmentation pathway of different types of compounds is of great interest in the identification of numerous compounds.

#### 3.4.1 Flavonoids analysis

**FIGURE 5:**
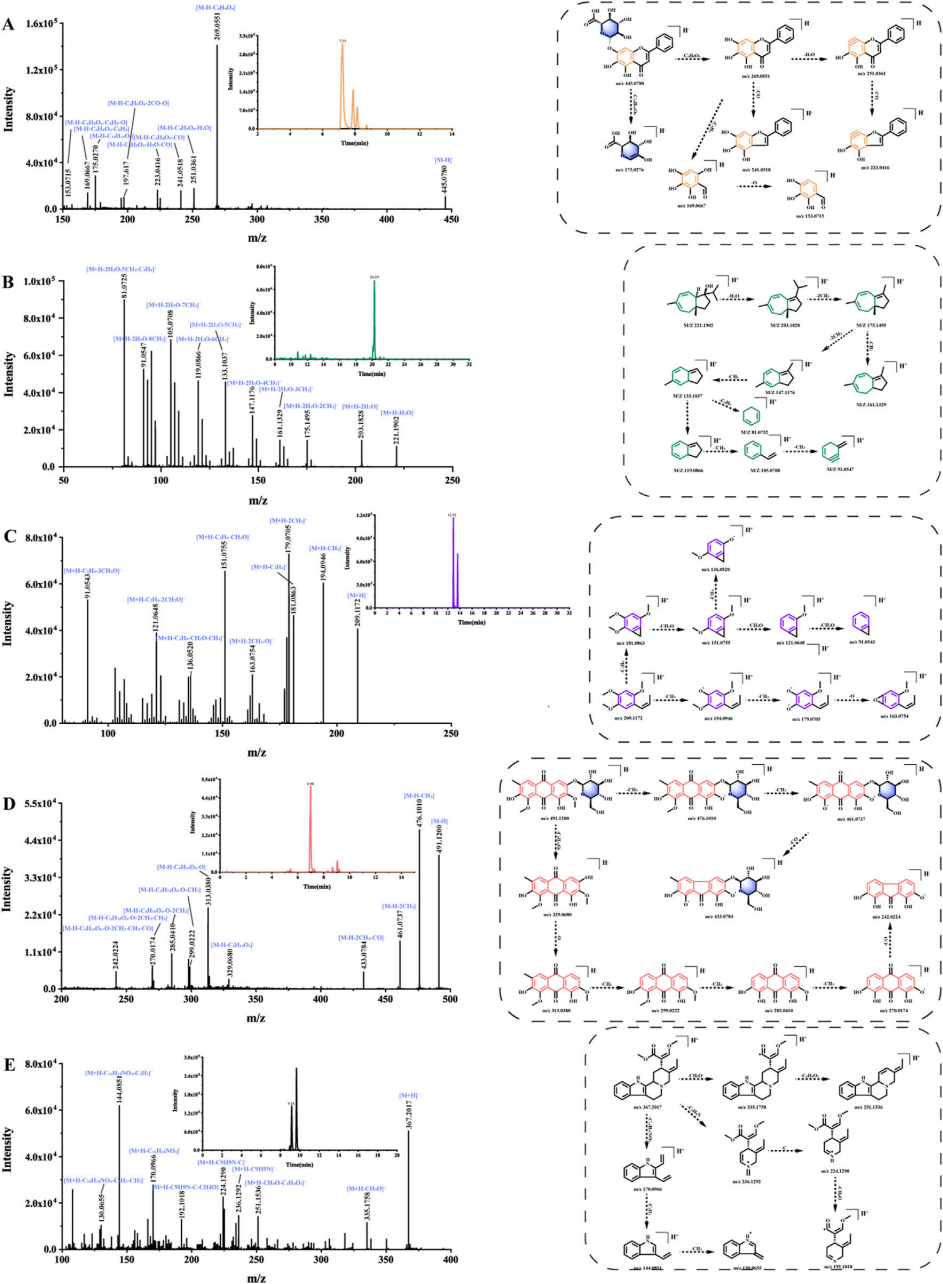
Fragmentation routes of the main components of ASDZL: **(A)** baicalin, **(B)** jaeschkeanadiol, **(C)** β-asarone, **(D)** geissoschizine methyl ether, and **(E)** glucoaurantio-obtusin.

There are 60 compounds of flavonoids in ASDZL, which are mainly from Scu. They mainly include flavonoid glycosides, flavonoids, isoflavonoids, flavonols, dihydroflavonoids, and polymethoxyflavonoids (PMFs) (Zhang et al., 2024b). Due to their structural diversity, flavonoids exhibit significant potential in anti-inflammatory and antioxidant properties (Ramanan et al., 2016; Wang et al., 2021). Flavonoid glycosides generally tend to lose glycosyl moieties during fragmentation pathways. Additionally, the classical RDA cleavages are characteristic fragmentation pathways for flavonoids. Furthermore, hydroxyl, methyl, or methoxy groups are usually attached to the flavonoid ring (A ring), resulting in the losses of the following neutral fragments: CH_3_ (−15 Da), CH_2_O (−30 Da), CO (−28 Da), H_2_O (−18 Da), and O (−16 Da), for example, baicalin (compound 107, t_R_ = 7.23 min), and its chemical formula is C_21_H_18_O_11_ ([M–H]^-^, *m/z* 445.0780) (Figure 5A). In the negative MS^2^ spectrum, the fragmentation ion of *m/z* 269.0551 [M–H–C_6_H_8_O_6_]^-^ was observed after losing glucuronic acid (GlcA, 176 Da). Continued losses of H_2_O (−18 Da) and CO (−28 Da) neutral fragments yielded *m/z* 251.0361 [M–H–C_6_H_8_O_6_–H_2_O]^-^ and *m/z* 223.0416 [M–H–C_6_H_8_O_6_–H_2_O–CO]^-^, respectively. Because of classical RDA cleavage, fragmentation ions at *m/z* 169.0667 were also detected in the MS^2^ spectrum. Fragmentation ions of *m/z* 269.0551 and *m/z* 169.0667 can be considered as diagnostic ions. Similarly, the fragment pattern of baicalin is the same as that of baicalein (compound 152, t_R_ = 10.02 min) (Supplementary Figure S4A). In addition, except compound 37, all other flavonoids exhibit characteristic fragment patterns. However, compound 37 was annotated by positive FBMN, and its fragment ions were consistent with the GNPS online library.

#### 3.4.2 Terpenoids analysis

Terpenoid compounds are mainly derived from Cur and Bup. There are six monoterpenes, 31 sesquiterpenes, and 13 triterpenes. Because of the presence of hydroxyl, methyl, and carbonyl groups in the structure of terpenoids, their MS^2^ spectrum showed the losses of CO (−28 Da), H_2_O (−18 Da), and CH_2_ (−14 Da) neutral molecules. In terms of terpenoids, sesquiterpenes are characteristic bioactive components from Cur (Zhao et al., 2023). For instance, under the positive FBMN workflow, jaeschkeanadiol (compound 234, t_R_ = 20.29 min) was annotated. Jaeschkeanadiol (Figure 5B) (C_15_H_24_O) yielded a quasi-molecular ion at *m/z* 203.1828 [M + H–H_2_O]^+^. The fragment ions at *m/z* 175.1495 [M + H–H_2_O–2CH_2_]^+^, *m/z* 161.1329 [M + H–H_2_O–3CH_2_]^+^, *m/z* 147.1176 [M + H–H_2_O–4CH_2_]^+^, *m/z* 133.1037 [M + H–H_2_O–5CH_2_]^+^, *m/z* 119.0866 [M + H–H_2_O–6CH_2_]^+^, *m/z* 105.0708 [M + H–H_2_O–7CH_2_]^+^, *m/z* 91.0547 [M + H–H_2_O–8CH_2_]^+^, and *m/z* 81.0725 [M + H–H_2_O–5CH_2_–C_4_H_4_]^+^ were yielded in its positive MS^2^ spectrum. The characteristic fragment pattern is due to the continuous losses of CH_2_ (−14 Da). In summary, 31 compounds exhibiting similar MS^2^ fragmentation patterns were identified as sesquiterpenes, four of which were further annotated *via* the FBMN analysis.

For monoterpenes, aucubin (Supplementary Figure S4B) (compound 10, t_R_ = 0.92 min) showed an excimer ion at *m/z* 345.1188 [M–H]^-^. In the negative ion MS^2^ spectrum, the successive losses of neutral fragments CH_2_O (−30 Da), O (−16 Da), and glucosyl residue (−162 Da) yield fragment ions at *m/z* 299.1147 [M–H–CH_2_O–O]^-^ and *m/z* 137.0609 [M–H–CH_2_O–O–C_6_H_10_O_5_]^-^. Then, the fragment ions at *m/z* 119.0446 [M–H–CH_2_O–O–C_6_H_10_O_5_–H_2_O]^-^, *m/z* 113.0248 [M–H–CH_2_O–O–C_6_H_10_O_5_–2C]^-^, and *m/z* 101.0247 [M–H–CH_2_O–O–C_6_H_10_O_5_–2H_2_O]^-^ referred to successive neutral losses of H_2_O (−18 Da) and carbon chain C_2_ (−24 Da). The MS^2^ spectrum also yielded fragment ions at *m/z* 179.0565 [M–H–C_9_H_10_O_3_]^-^ and *m/z* 89.0249 [M–H–2CH_2_O–O–C_6_H_10_O_5_–H_2_O]^-^. Likewise, compounds 2, 4, 10, and 42 generated similar MS^2^ fragment ions at *m/z* 179.0565 and *m/z* 89.0249, which were considered diagnostic ions.

Triterpenoids in ASDZL include oleanane-type pentacyclic triterpenoids and ursane-type tetracyclic triterpenoids, for example, ursolic acid (Supplementary Figure S4C) (compound 241, t_R_ = 23.52 min) from ursane-type tetracyclic triterpenoids, and its formula was C_30_H_48_O_3_ ([M + H]^+^, *m/z* 457.3677). In the positive ion mode, fragmentation ions of *m/z* 439.3579, *m/z* 411.3607, and *m/z* 393.3513 were detected owing to successive losses of H_2_O (−18 Da) and CO_2_ (−28 Da). According to literature reports, ursolic acid may have three RDA cleavage patterns. In this study, based on characteristic RDA cleavage, fragment ions (diagnostic ions) at *m/z* 249.1352, m*/z* 203.1791, and *m/z* 191.1795 were observed in its MS^2^ spectrum (Xu et al., 2025). Similarly, compounds 225, 239, 242, and 243 showed similar MS^2^ fragment patterns. However, compounds 147, 164, 173, 193, 199, 200, 204, and 208, identified as oleanane-type pentacyclic triterpenoids, were prone to losing glycoside units at glycoside (−162 Da) or arabinose (−146 Da) in the negative ion mode.

#### 3.4.3 Phenylpropanoids analysis

In this study, 24 phenylpropanoids were identified in ASDZL. Some phenylpropanoids were observed to lose neutral molecules, including H_2_O (−18 Da), CO_2_ (−44 Da), CH_3_ (−15 Da), CH_2_O (−30 Da), and CO (−28 Da). β-Asarone (Figure 5C) (compound 188, t_R_ = 12.92 min) exhibited a [M + H]^+^ quasi-molecular ion at *m/z* 209.1172. The product ions at *m/z* 194.0946 [M + H–CH_3_]^+^, *m/z* 181.0836 [M + H–CH_2_O]^+^, *m/z* 179.0705 [M + H–2CH_3_]^+^, *m/z* 163.0754 [M + H–2CH_3_–O]^+^, *m/z* 151.0755 [M + H–2CH_2_O]^+^, *m/z* 136.0520 [M + H–2CH_2_O–CH_3_]^+^, *m/z* 121.0648 [M + H–3CH_2_O]^+^, and *m/z* 91.0543 [M + H–4CH_2_O]^+^ were detected in the positive MS^2^ spectrum. According to literature reports, in addition to β-asarone, there is also its isomer α-asarone, and β-asarone has a shorter retention time (Wei et al., 2022). Therefore, compound 188 was identified as β-asarone, and compound 195 was identified as α-asarone. In addition, four compounds (38, 55, 72, and 95) showed losses of glucosyl residue (−162 Da) or rutinoside residue (−308 Da) in the MS^2^ spectrum. The remaining phenylpropanoids exhibited similar neutral losses.

#### 3.4.4 Alkaloids analysis

The alkaloids are predominantly derived from Unc. Because of the lone electron pair on the nitrogen atom, these alkaloids are readily protonated under positive-ion electrospray ionization conditions, leading to enhanced peak symmetry and detection sensitivity. Unc primarily contains monoterpene indole alkaloids, which are divided into tetracyclic and pentacyclic monoterpene indole alkaloids. Indole alkaloids possess complicated structures. According to literature reports, they are generally identified based on the open-ring cleavage of their parent nucleus and the fragments information after the loss of the nitrogen bridge ring neutral molecule (Pan et al., 2015), for example, geissoschizine methyl ether (Figure 5D) (compound 144, t_R_ = 9.16 min) from tetracyclic monoterpene indole alkaloids. The positive ion MS^2^ spectrum yielded a parent ion at [M + H]^+^ *m/z* 367.2017. The product ions at *m/z* 335.1758 and *m/z* 251.1537 were found owing to neutral losses of CH_4_O and C_4_H_4_O_2_. The most characteristic fragmentation pathway for monoterpene indole alkaloids was the preferable cleavage of ring C, which produces diagnostic ions. In this way, fragment ions at *m/z* 236.1292 [M + H–C_9_H_9_N]^+^ and *m/z* 170.0966 [M + H–C_10_H_15_NO_3_]^+^ were observed through the cleavage of ring C. Then, the product ions were found at *m/z* 224.1292 [M + H–C_9_H_9_N-C]^+^, *m/z* 192.1080 [M + H–C_9_H_9_N–C–CH_4_O]^+^, *m/z* 144.0851 [M + H–C_10_H_15_NO_3_–C_2_H_2_]^+^, and *m/z* 130.0655 [M + H–C_10_H_15_NO_3_–C_2_H_2_–CH_2_]^+^. Furthermore, nine compounds (94, 96, 108, 109, 115, 140, 144, 148, and 155) were annotated in positive FBMN.

#### 3.4.5 Anthraquinones analysis

Anthraquinones are characteristic bioactive components from Sen. Anthraquinones were prone to losing neutral molecules, including H_2_O (−18), O (−16 Da), CH_3_ (−15 Da), and CO (−28 Da). Additionally, anthraquinone glycosides usually presented glycosyl moieties in their MS^2^ spectrum, for example, glucoaurantio-obtusin (Figure 5E) (compound 100, t_R_ = 6.98 min), whose molecular formula was C_23_H_24_O_12_ ([M–H]^-^, *m/z* 491.1200). The fragment ions at *m/z* 476.1010 [M–H–CH_3_]^-^, *m/z* 461.0737 [M–H–2CH_3_]^-^, and *m/z* 433.0784 [M–H–2CH_3_–CO]^-^ emerged due to consecutive neutral losses. The fragment ions at *m/z* 329.0680 were detected due to neutral loss of glucosyl residue (−162 Da). Other product ions at *m/z* 313.0380, m*/z* 299.0222, m*/z* 285.0410, m*/z* 270.0174, and m*/z* 242.0224 referred to consecutive neutral losses of O (−16 Da), CH_2_ (−14 Da), CH_3_ (−15 Da), and CO (−28 Da). A total of 18 compounds (58, 85, 92, 100, 126, 135, 136, 141, 153, 163, 167, 176, 182, 194, 196, 203, 218, and 226) were identified as anthraquinones owing to similar MS^2^ fragment behaviors.

#### 3.4.6 Other types of analysis

In addition to the above five main types of compounds, ASDZL also contains 16 phenylethanoid glycosides, 13 phenolic acids, nine xanthones, nine oligosaccharides, eight phthaleins, nine naphthopyrones, four organic acids, four aromatic aldehydes, and two diarylheptanoids. Interestingly, owing to the unique chemical structures of diarylheptanoids, they have been extensively studied. These compounds exhibit significant anti-inflammatory, antioxidant, and anticancer effects (Sudarshan et al., 2024). Phenylethanoid glycosides are formed by the formation of a glycosidic bond between phenethyl alcohol and β-D-glucopyranose. Forsythoside A (Supplementary Figure S4D) (compound 63, t_R_ = 9.16 min) generated a parent ion at *m/z* 623.1984 [M–H]^-^. The fragment ions at *m/z* 461.1665 and *m/z* 443.1542 were detected, owing to consecutive neutral losses of glucosyl residue (−162 Da) and H_2_O (−18 Da). Then, the product ions at *m/z* 179.0351 [M–H–C_20_H_18_O_11_]^-^, *m/z* 179.0351 [M–H–C_20_H_18_O_11_–O]^-^, and *m/z* 135.0452 [M–H–C_20_H_18_O_11_–CO_2_]^-^ were found in the MS^2^ spectrum. The remaining eight phenylethanoid glycosides showed similar mass fragment patterns. Oligosaccharides are the main characteristic components of Pol. Glucoses or rhamnoses, which are mainly based on sucrose as a common core, are connected by different forms of glycosidic bonds to form oligosaccharides, which then form esters with organic acid components (Zhang et al., 2023). For instance, tenuifoliside C (Supplementary Figure S4E) (compound 124,t_R_ = 8.25 min) generated an excimer ion at *m/z* 767.2398 [M–H]^-^. The product ions are generated at *m/z* 529.1556 [M–H–C_12_H_14_O_5_]^-^, *m/z* 341.1987 [M–H–C_23_H_22_O_8_]^-^, *m/z* 325.0892 [M–H–C_23_H_22_O_8_–O]^-^, *m/z* 295.0818 [M–H–C_23_H_22_O_8_–O–CH_2_O]^-^, *m/z* 265.0721 [M–H–C_23_H_22_O_8_–O–2CH_2_O]^-^, *m/z* 237.0764 [M–H–C_23_H_30_O_14_]^-^, *m/z* 223.0617 [M–H–C_23_H_30_O_14_–CH_2_]^-^, and *m/z* 205.0507 [M–H–C_23_H_30_O_14_–CH_2_–CO]^-^. Likewise, the remaining eight oligosaccharides showed similar mass fragment patterns.

### 3.5 Identification of ASDZL-related constituents *in vivo*

Research shows that when TCMs are taken orally, and their primary metabolic pathways involve hydrolyzation, methylation, oxidation, sulfation, glucuronidation, and reduction reactions (Guo et al., 2022). The identification of ASDZL components *in vivo* is likely to be compromised by endogenous interference from complex biological matrices. To address this, extracted ion chromatograms (XICs) can be generated from MS data to improve detection sensitivity and simplify data processing. In this study, the mass spectrum analysis was performed in positive and negative ion modes using UHPLC-HRMS. The ASDZL-related constituents *in vivo* was based on molecular formula, retention time, accurate mass and fragment ions information. The prototype components in the rat plasma and cerebrum samples were identified using the XIC function in PeakView™ software. Subsequently, MetabolitePilot™ software was used to predict potential metabolites. The predicted metabolites were further compared with prototype compounds and data in the literature using the XIC function in PeakView™ software. In addition, these potential metabolites can be screened using DIF and NLF strategies. Finally, based on literature reports and the identified prototypes and metabolites, the metabolic pathways of the main types of chemical components in ASDZL were elucidated. In the negative ion mode, [M–H]^−^ was detected, whereas in the positive ion mode, [M + H]^+^ was observed. Our analysis showed that 50 constituents were identified from plasma samples, whereas these components were not observed in the blank plasma samples. In addition, 16 compounds were found in the cerebrum samples of the ASDZL-administered rats that were not identified in the blank cerebrum samples (Supplementary Figure S6). A total of 50 prototypes were detected *in vivo*. In addition, 16 compounds (Supplementary Table S4) were found to be shared in the plasma and cerebrum samples, indicating that a significant portion of TCM components cannot cross the blood–brain barrier to reach the target in the brain. The interaction between complex Chinese herbal medicine constituents and the gut microbiota is a new target. Therefore, the brain–gut axis may serve as a bridge for studying the interaction between TCMs and the brain (Feng et al., 2019; Feng et al., 2023).

#### 3.5.1 Prototype and metabolite components in bio-samples

**FIGURE 6:**
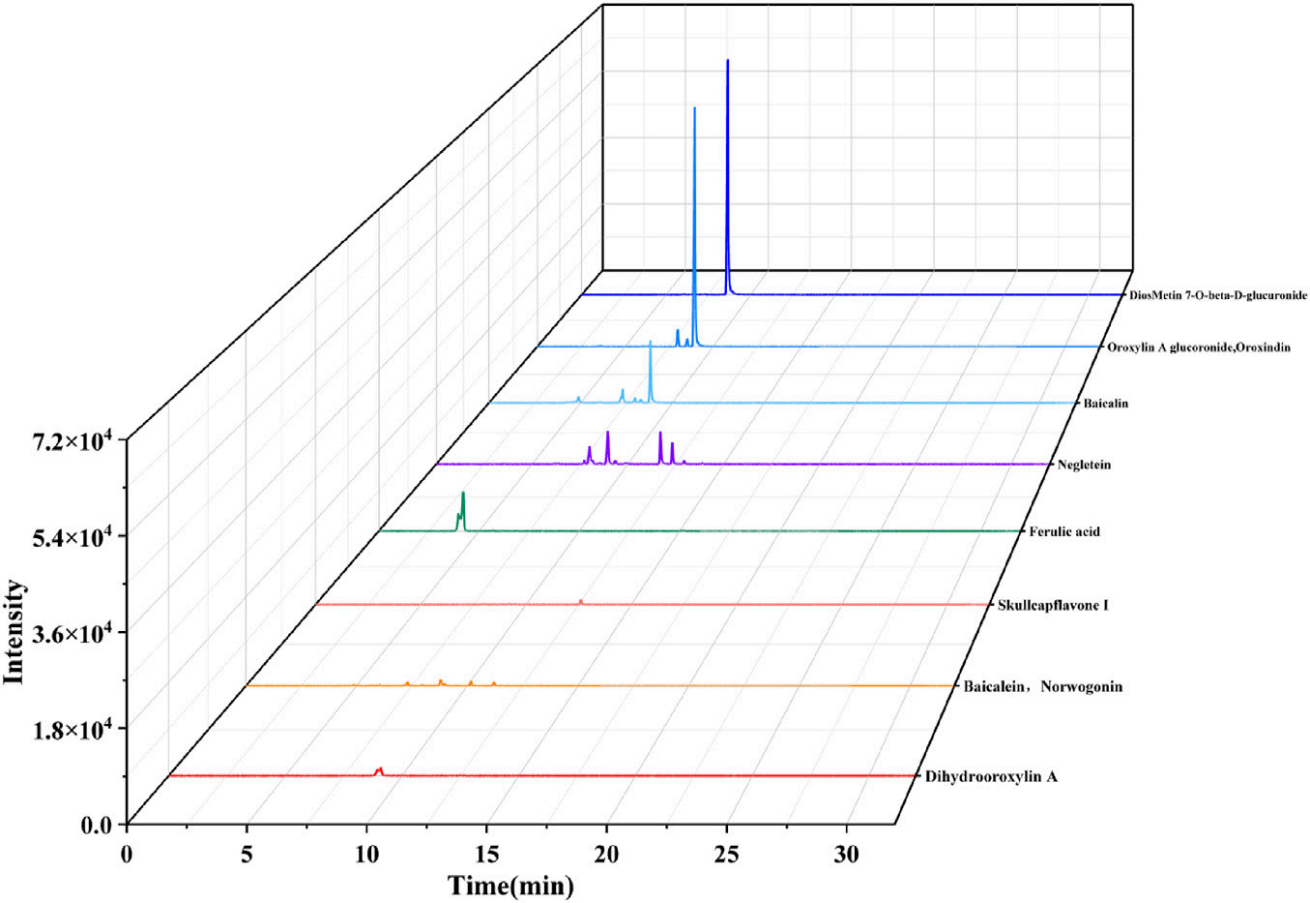
Extraction of ion chromatograms of 10 prototypes with relative high intensity from plasma samples.

There were 50 prototypes, four of which were confirmed by reference standards, including ferulic acid, baicalin, rhynchophylline, and oroxindin. The prototypes included 22 flavonoids, 11 terpenoids, four alkaloids, four phenolic acids, four phenylpropanoids, one anthraquinone, one naphthopyrone, one xanthones, one phthalein, and 1 organic acid. Screening analysis of the XIC diagrams of the 50 prototypes revealed that 10 compounds (P5, P17, P22–P26, P33, P35, and P39) had relatively high intensity, as shown in Figure 6. Baicalin, baicalein, oroxindin, and wogonin are characteristic bioactive components from Scu (Zhang et al., 2025). These four compounds have been reported to exhibit significant anti-inflammatory and antiviral activities through *in vitro* experiments and molecular docking studies (Liu et al., 2023). Baicalin and baicalein also have significant antioxidant capacity. However, the low bioavailability of baicalin and oroxindin limits their application (Pan et al., 2021). After oral administration, baicalin and oroxindin are absorbed in small amounts in the stomach and small intestine. In the colon, baicalin and oroxindin, acting as prodrugs, can be hydrolyzed by microbial *β*-glucuronidase into their aglycones, baicalein, and wogonin (Wang et al., 2012; Noh et al., 2016). This metabolic conversion enhances their bioavailability, allowing better colonic absorption and generating pharmacological effects. Furthermore, baicalin can alleviate neuroinflammation by inhibiting Toll-like receptor (Guo et al., 2019). Baicalin and baicalein can significantly inhibit the enzymatic action of MAO-B and then alleviate neuronal suppression mediated by astrocytic GABA (Cho et al., 2024). Wogonin attenuates LPS + ATP-induced inflammatory damage by inhibiting the NLRP3/GSDMD pyroptosis pathway and regulating the CD39 purinergic pathway (Wang et al., 2024). Ferulic acid exhibits multiple pharmacological activities, including antioxidant, anti-inflammatory, antitumor, antidepressant, and neuroprotective effects. In addition, its antidepressant and neuroprotective properties have been validated in both *in vivo* and *in vitro* studies (Dong and Huang, 2022). Clinical applications of ferulic acid and its derivatives remain limited, mainly due to insufficient understanding of their mechanisms of action. Additional studies are needed to elucidate their molecular targets and signaling pathways. Except ferulic acid, which is a phenolic acid compound, the rest are flavonoid compounds. This indicates that flavonoid compounds are the main biological constituents absorbed following oral administration of ASDZL extract. The BPC diagrams of ASDZL in the plasma and cerebrum samples in the positive ion mode and the negative ion mode are shown in Supplementary Figure S6. The identification results and related mass spectrum information of prototypes and 60 metabolites are shown in Tables 1, 2, including 34 flavonoids, 18 phenolic acids, six phenylpropanoids, one terpenoid, and one anthraquinone.

**TABLE 1:** Absorbed constituents in plasma samples after oral administration of ASDZL (P50).

NO.	RT (min)	Identification	Formula	Selected ion	Theoretical mass (Da)	Measured mass (Da)	Error (ppm)	MS/MS fragments	Plasma	Cerebrum
P1	0.77	8-Epiloganic acid	C_16_H_24_O_10_	[M–H]^-^	375.1297	375.1296	−0.21	213.0770, 169.0885, 151.0760, 125.0610	**+**	**-**
P2	1.33	Caffeic acid	C_9_H_8_O_4_	[M–H]^-^	179.0350	179.0353	1.77	135.0458, 89.0394	**+**	**-**
P3	1.67	p-Coumaric acid	C_9_H_8_O_3_	[M–H]^-^	163.0401	163.0399	−1.27	119.0510. 93.1455	**+**	**-**
P4	1.92	Protocatechuic acid	C_7_H_6_O_4_	[M–H]^-^	153.0193	153.0190	−1.97	135.0091, 109.0300, 91.0192	**+**	**-**
P5	3.86	Ferulic acid*	C_10_H_10_O_4_	[M + H]^+^	195.0652	195.0652	0.06	177.0543, 163.0392, 145.0281, 117.0334, 89.0385	**+**	**+**
P6	4.54	Zedoalactone C	C_15_H_22_O_4_	[M + H]^+^	267.1591	267.1595	1.39	249.1497, 231.1370, 213.1273, 203.1432, 185.1320, 147.1160, 133.1010	**+**	**+**
P7	4.71	Chrysin 6-C-arabinoside 8-C-glucoside	C_26_H_28_O_13_	[M + H]^+^	549.1603	549.1612	1.62	495.1340, 465.1200, 363.0874	**+**	**-**
P8	5.40	5,7-Dihydroxy-2-phenyl-6-[3,4,5-trihydroxy-6-(hydroxymethyl) oxan-2-YL]-8-(3,4,5-trihydroxyoxan-2-YL) chromen-4-one	C_26_H_28_O_13_	[M + H]^+^	549.1603	549.1598	−0.79	531.1546, 483.1377	**+**	**-**
P9	5.46	Zedoalactone A	C_15_H_22_O_4_	[M + H]^+^	267.1591	267.1580	−4.17	249.1479, 231.1374, 213.1290, 185.1377	**+**	**-**
P10	6.47	Hispidulin 7-glucuronide	C_22_H_20_O_12_	[M + H]^+^	477.1028	477.1050	4.74	301.0708, 286.0495	**+**	**-**
P11	6.74	Isocorynoxeine	C_22_H_26_N_2_O_4_	[M + H]^+^	383.1965	383.1959	−1.70	267.1511, 160.0758	**+**	**-**
P12	6.77	Corynoxeine	C_22_H_26_N_2_O_4_	[M + H]^+^	383.1965	383.1968	0.81	160.0751	**+**	**-**
P13	7.13	3,4,5-Trihydroxy-6-[5-hydroxy-2-(4-hydroxy-3-methoxyphenyl)-3-methoxy-4-oxochromen-7-yl] oxyoxane-2-carboxylic acid	C_23_H_22_O_13_	[M–H]^-^	505.0988	505.0984	−0.70	329.0672, 314.0440, 299.0199	**+**	**-**
P14	7.18	5.2′,6′-Trihydroxy-7,8-dimethoxyflavone 2′-glucuronide	C_23_H_22_O_13_	[M-H]^-^	505.0988	505.0986	−0.36	329.0671, 314.0435, 299.0195	**+**	**-**
P15	7.25	Cynaroside	C_21_H_20_O_11_	[M–H]^-^	447.0933	447.0939	1.30	271.0608, 256.0383	**+**	**-**
P16	7.33	Rhynchophylline*	C_22_H_28_N_2_O_4_	[M + H]^+^	385.2122	385.2146	6.22	353.1868, 269.1672, 160.0763	**+**	**-**
P17	7.34	Baicalin*	C_21_H_18_O_11_	[M–H]^-^	445.0776	445.0777	0.22	269.0461, 251.0346, 241.0511, 223.0406, 175.0250, 169.0661	**+**	**+**
P18	7.59	3,4,5-Trimethoxycinnamic acid	C_12_H_14_O_5_	[M + H]^+^	239.0914	239.0914	−0.19	206.0570, 193.0841, 191.0338, 178.0624, 163.0391	**+**	**-**
P19	7.86	Viscidulin II	C_17_H_14_O_7_	[M + H]^+^	331.0812	331.0812	−0.06	316.0656, 298.0504, 270.0525, 242.0575	**+**	**-**
P20	8.32	Glychionide A	C_21_H_18_O_11_	[M-H]^-^	445.0776	445.0783	1.59	269.0461, 241.0506, 225.0551, 197.0608	**+**	**-**
P21	8.53	Chrysin-7-O-glucuronide	C_21_H_18_O_10_	[M–H]^-^	429.0827	429.0836	1.98	253.0509, 175.0267, 113.0245, 99.0082, 85.0304	**+**	**-**
P22	8.57	Oroxylin A glucuronide	C_22_H_20_O_11_	[M–H]^-^	459.0933	459.0930	−0.69	283.0618, 268.0383	**+**	**-**
P23	8.64	DiosMetin 7-O-beta-D-glucuronide	C_22_H_20_O_12_	[M + H]^+^	477.1028	477.1040	2.60	301.0779, 286.0511	**+**	**+**
P24	8.80	Norwogonin	C_15_H_10_O_5_	[M + H]^+^	271.0601	271.0611	3.85	253.0492, 225.0533, 169.0118, 141.0676, 123.0080	**+**	**+**
P25	8.86	Dihydrooroxylin A	C_16_H_14_O_5_	[M + H]^+^	287.0914	287.0917	1.03	183.0292, 168.0050, 140.0095, 131.0487	**+**	**-**
P26	9.00	Oroxindin*	C_22_H_20_O_11_	[M–H]^-^	459.0933	459.0934	0.19	283.0626, 268.0384	**+**	**+**
P27	9.14	1-Hydroxy-3,7-dimethoxyxanthone	C_15_H_12_O_5_	[M + H]^+^	273.0758	273.0765	2.69	255.0747, 227.0673	**+**	**-**
P28	9.15	Tectoridin	C_22_H_22_O_11_	[M–H]^-^	461.1089	461.1081	−1.83	285.0773, 175.0253, 165.9908, 137.9956	**+**	**-**
P29	9.25	Geissoschizine methyl ether	C_22_H_26_N_2_O_3_	[M + H]^+^	367.2016	367.2023	1.79	335.1782, 224.1286, 170.0953, 144.0794	**+**	**-**
P30	9.33	Curcumin C	C_15_H_16_O_3_	[M + H]^+^	245.1172	245.1167	−2.10	229.0466, 199.0348, 181.0748	**+**	**-**
P31	10.08	5.2′,6′-Trihydroxy-6,7,8-trimethoxyflavone	C_18_H_16_O_8_	[M + H]^+^	361.0918	361.0906	−3.41	346.0686, 331.0447, 313.0347	**+**	**-**
P32	10.10	Curdionolide B	C_15_H_20_O_3_	[M + H]^+^	249.1485	249.1484	−0.31	231.1378, 213.1272, 203.1422, 185.1321, 161.0991, 147.0803	**+**	**-**
P33	10.18	Baicalein	C_15_H_10_O_5_	[M + H]^+^	271.0601	271.0605	1.33	253.0532,225.0542,169.0649,123.0074	**+**	**+**
P34	10.78	Curcolone	C_15_H_18_O_3_	[M + H]^+^	247.1329	247.1339	4.19	229.1234, 211.1103, 201.0803, 183.1171, 143.0845, 129.0206	**+**	**-**
P35	11.69	Negletein	C_16_H_12_O_5_	[M + H]^+^	285.0758	285.0763	1.77	270.0540, 168.0063, 140.0105	**+**	**-**
P36	11.73	Curdione	C_15_H_24_O_2_	[M + H]^+^	237.1849	237.1851	0.94	219.1343, 201.1609, 175.1407, 159.1173, 145.1027	**+**	**-**
P37	12.31	β-Asarone	C_12_H_16_O_3_	[M + H]^+^	209.1172	209.1178	2.73	181.1217, 179.1089, 121.9462, 91.0546	**+**	**-**
P38	12.32	Wogonin	C_16_H_12_O_5_	[M + H]^+^	285.0758	285.0765	2.71	270.0531, 252.0428, 242.0583, 179.0492, 151.0539	**+**	**+**
P39	12.61	Skullcapflavone I	C_17_H_14_O_6_	[M + H]^+^	315.0863	315.0868	1.50	300.0628, 285.0397, 282.0521, 257.0438	**+**	**+**
P40	12.75	Methyl eugenol	C_11_H_14_O_2_	[M + H]^+^	179.1067	179.1065	−0.62	151.0753, 121.0622, 107.0481, 91.0536	**+**	**+**
P41	12.92	Oroxylin A	C_16_H_12_O_5_	[M + H]^+^	285.0758	285.0758	0.22	270.0523, 168.0051	**+**	**+**
P42	13.86	Obtusifolin	C_16_H_12_O_5_	[M + H]^+^	285.0758	285.0759	0.68	270.0510, 253.0404, 242.0579, 225.0548, 211.0752	**+**	**-**
P43	13.95	α-Asarone	C_12_H_16_O_3_	[M + H]^+^	209.1172	209.1175	1.28	181.1204, 179.1075, 165.0722, 135.1147, 121.1031, 91.0538	**+**	**+**
P44	14.32	Procurcumenol	C_15_H_22_O_2_	[M + H]^+^	235.1693	235.1692	−0.24	217.1569, 189.1635, 175.1105, 161.0956, 133.1003, 119.0846	**+**	**-**
P45	15.82	Toralactone	C_15_H_12_O_5_	[M + H]^+^	273.0758	273.0760	1.08	255.0642, 227.0700, 212.0465, 184.0515	**+**	**+**
P46	16.55	Oxyphyllanene B	C_12_H_14_O_2_	[M + H]^+^	191.1067	191.1069	1.29	173.0934, 163.1087, 145.0981, 131.0888	**+**	**+**
P47	16.88	Saucernetin	C_22_H_28_O_5_	[M + H]^+^	373.2010	373.2016	1.83	235.1309, 217.1207, 202.0976, 179.1057, 165.0887, 151.0754	**+**	**-**
P48	19.05	Calamenene	C_15_H_22_	[M + H]^+^	203.1794	203.1797	1.39	203.1121, 147.1136, 133.1025, 119.0916	**+**	**-**
P49	19.49	4,7-Dihydroxy-3-butylphthalide	C_12_H_14_O_4_	[M + H]^+^	223.0965	223.0962	−1.30	207.0318, 191.0009, 149.0235	**+**	**+**
P50	20.44	Eremophilene	C_15_H_24_	[M + H]^+^	205.1951	205.1953	0.94	149.0234, 135.1174, 121.1006, 107.0838, 93.0698	**+**	**+**

Note: P: prototype component; * confirmed by comparing with the reference standards; + means detectable, - means undetectable, RT(min) means retention time(min).

**TABLE 2:** Metabolites in plasma samples after oral administration of ASDZL (60 M).

NO.	Rt (min)	Identification	Formula	Selected ion	Theoretical mass (Da)	Measured mass (Da)	Error (ppm)	MS/MS fragments	Types
M1	0.73	Caffeic acid-2O	C_9_H_8_O_2_	[M–H]^-^	147.0452	147.0454	1.50	145.0497, 103.0576, 89.0246	Phenolic acids
M2	0.76	Ferulic acid+2H	C_10_H_12_O_4_	[M + H]^+^	197.0808	197.0805	−1.77	179.0718, 161.0596, 151.0388, 135.0446, 133.0648, 105.0692, 91.0540	Phenolic acids
M3	0.82	Protocatechuic acid + SO_3_+CH_3_	C_8_H_8_O_7_S	[M–H]^-^	246.9918	246.9918	−0.07	167.0354, 152.0118, 123.0453, 108.0220	Phenolic acids
M4	0.86	Caffeic acid + SO_3_+2H	C_9_H_10_O_7_S	[M–H]^-^	261.0075	261.0074	−0.25	217.0182, 181.0516, 137.0614	Phenolic acids
M5	1.00	Caffeic acid+2H	C_9_H_10_O_4_	[M–H]^-^	181.0506	181.0507	0.23	163.0408, 135.0457, 119.0507	Phenolic acids
M6	1.01	Protocatechuic acid-CO_2_+SO_3_	C_6_H_6_O_5_S	[M–H]^-^	188.9863	188.9865	1.18	109.0310, 91.0192	Phenolic acids
M7	1.25	Ferulic acid-CH_2_	C_9_H_8_O_4_	[M + H]^+^	181.0495	181.0501	3.08	163.0391, 145.0274, 141.0728, 135.0449, 117.0344, 105.0701, 89.0382	Phenolic acids
M8	1.25	Caffeic acid + C_6_H_8_O_6_	C_15_H_16_O_10_	[M–H]^-^	355.0671	355.0675	1.31	179.0358, 135.0457	Phenolic acids
M9	1.35	Caffeic acid + SO_3_	C_9_H_8_O_7_S	[M–H]^-^	258.9918	258.9924	2.45	179.0354, 135.0456	Phenolic acids
M10	1.39	Caffeic acid + CH_2_	C_10_H_10_O_4_	[M–H]^-^	193.0506	193.0509	1.15	178.0255, 134.0379	Phenolic acids
M11	1.42	Ferulic acid + SO_3_	C_10_H_10_O_7_S	[M–H]^-^	273.0075	273.0073	−0.45	193.0512, 178.0279, 149.0615, 134.0380	Phenolic acids
M12	1.49	Caffeic acid + SO_3_+CH_2_	C_10_H_10_O_7_S	[M–H]^-^	273.0075	273.0073	−0.54	193.0512, 178.0274, 149.0609, 134.0377	Phenolic acids
M13	1.62	Caffeic acid-O	C_9_H_8_O_3_	[M–H]^-^	163.0401	163.0399	−0.90	145.8898, 119.0507	Phenolic acids
M14	1.67	Ferulic acid-CH_2_-O	C_9_H_8_O_3_	[M–H]^-^	163.0401	163.0399	−1.27	119.0510, 93.1455	Phenolic acids
M15	1.67	Caffeic acid + SO_3_-O	C_9_H_8_O_6_S	[M–H]^-^	242.9969	242.9965	−1.60	163.0410, 119.0508	Phenolic acids
M16	1.93	Ferulic acid + C_6_H_8_O_6_	C_16_H_18_O_10_	[M–H]^-^	369.0827	369.0820	−2.08	193.0510, 178.0271, 134.0377	Phenolic acids
M17	2.15	Zedoalactone C+2H	C_15_H_24_O_4_	[M + H]^+^	269.1747	269.1735	−4.63	251.1648, 233.1556, 215.1459, 203.1461	Terpenoids
M18	4.08	Ferulic acid + CH_2_+2H	C_11_H_14_O_4_	[M + H]^+^	211.0965	211.0964	−0.62	181.1248, 179.0670, 149.0248, 131.0855, 115.0516, 105.0738, 91.0540	Phenolic acids
M19	4.45	Baicalein-O+2H	C_15_H_12_O_4_	[M + H]^+^	257.0808	257.0801	−2.81	163.0395, 151.1130, 135.0441, 123.0439	Flavonoids
M20	6.09	Oroxindin + O	C_22_H_20_O_12_	[M + H]^+^	477.1028	477.1024	−0.75	301.0705	Flavonoids
M21	6.11	Baicalin + C_6_H_8_O_6_	C_27_H_26_O_17_	[M–H]^-^	621.1097	621.1095	−0.37	445.0774, 269.0457	Flavonoids
M22	6.12	Norwogonin + C_6_H_8_O_6_	C_21_H_18_O_11_	[M–H]^-^	445.0776	445.0755	−4.85	269.0455	Flavonoids
M23	6.16	Glychionide A + C_6_H_8_O_6_	C_27_H_26_O_17_	[M–H]^-^	621.1097	621.1104	1.05	445.0779, 269.0459	Flavonoids
M24	6.19	Baicalin+2O+2H	C_21_H_20_O_13_	[M + H]^+^	481.0977	481.0975	−0.27	305.0663, 290.0445	Flavonoids
M25	6.37	Oroxindin + C_6_H_8_O_6_	C_28_H_28_O_17_	[M + H]^+^	637.1399	637.1416	2.54	461.1085, 285.0757	Flavonoids
M26	6.44	Baicalin + C_6_H_8_O_6_+CH_2_	C_28_H_28_O_17_	[M–H]^-^	635.1254	635.1253	−0.17	459.0914, 283.0619, 268.0382	Flavonoids
M27	6.49	Ferulic acid-O+2H	C_10_H_12_O_3_	[M + H]^+^	181.0859	181.0866	3.62	151.0371, 137.0577, 123.0797, 107.0866, 105.0356, 89.0392	Phenolic acids
M28	6.77	Dihydrooroxylin A + C_6_H_8_O_6_	C_22_H_22_O_11_	[M + H]^+^	461.1089	461.1096	1.41	285.0786, 270.0537, 252.0435	Flavonoids
M29	6.79	DiosMetin 7-O-beta-D-glucuronide + C6H8O6	C_28_H_28_O_18_	[M + H]^+^	653.1348	653.1369	3.20	477.1042, 301.0715	Flavonoids
M30	7.25	Tectoridin-CH_2_	C_21_H_20_O_11_	[M–H]^-^	447.0933	447.0939	1.30	271.0608, 256.0383	Flavonoids
M31	7.33	Baicalin + CH_2_+O	C_22_H_20_O_12_	[M–H]^-^	475.0882	475.0879	−0.72	299.0570, 284.0351	Flavonoids
M32	7.95	Skullcapflavone I + C_6_H_8_O_6_	C_23_H_22_O_12_	[M + H]^+^	491.1184	491.1209	5.17	315.0875, 300.0621, 285.0344	Flavonoids
M33	8.02	Wogonin + C_6_H_8_O_6_	C_22_H_20_O_11_	[M–H]^-^	459.0933	459.0938	1.04	283.0613, 268.0382	Flavonoids
M34	8.06	Baicalin + CH_2_	C_22_H_20_O_11_	[M–H]^-^	459.0933	459.0930	−0.66	283.0620, 268.0386	Flavonoids
M35	8.24	Glychionide A + CH_2_	C_22_H_20_O_11_	[M–H]^-^	459.0933	459.0924	−1.91	283.0615, 268.0380, 240.0427	Flavonoids
M36	8.45	Glychionide A-C_6_H_8_O_6_-O	C_15_H_10_O_4_	[M + H]^+^	255.0652	255.0654	0.99	199.0734, 181.1005, 153.0183	Flavonoids
M37	8.50	Glychionide A-O	C_21_H_18_O_10_	[M–H]^-^	429.0827	429.0811	−3.71	253.0512	Flavonoids
M38	8.64	Baicalein + CH_2_+O	C_16_H_12_O_6_	[M + H]^+^	301.0707	301.0715	2.66	286.0472, 183.9997, 155.9957, 127.0066	Flavonoids
M39	8.68	Baicalein+2H	C_15_H_12_O_5_	[M + H]^+^	273.0758	273.0749	−3.06	229.0489, 168.8711	Flavonoids
M40	8.69	Viscidulin II + CH_2_	C_18_H_16_O_7_	[M + H]^+^	345.0965	345.0965	0.00	330.0717, 312.0627, 284.0689, 266.0647	Flavonoids
M41	8.71	Chrysin-7-O-glucuronide-C_6_H_8_O_6_+SO_3_	C_15_H_10_O_7_S	[M–H]^-^	333.0075	333.0088	3.95	253.0515, 225.0562	Flavonoids
M42	9.03	Norwogonin-O	C_15_H_10_O_4_	[M + H]^+^	255.0652	255.0659	2.72	227.0705, 209.0576, 199.0745, 181.0635, 153.0697	Flavonoids
M43	9.06	DiosMetin 7-O-beta-D-glucuronide-O	C_22_H_20_O_11_	[M–H]^-^	459.0933	459.0934	0.24	283.0620, 268.0387, 175.0253	Flavonoids
M44	9.12	Baicalin-O	C_21_H_18_O_10_	[M–H]^-^	429.0827	429.0841	3.17	253.0509	Flavonoids
M45	9.20	Viscidulin II-O	C_17_H_14_O_6_	[M + H]^+^	315.0863	315.0861	−0.53	300.0435, 285.0328, 269.0952	Flavonoids
M46	9.21	Oroxindin + CH_2_+O	C_23_H_22_O_12_	[M + H]^+^	491.1184	491.1181	−0.70	315.0863, 300.0624, 285.0403	Flavonoids
M47	9.48	Methyl eugenol-2CH_2_	C_9_H_10_O_2_	[M + H]^+^	151.0754	151.0747	−4.19	107.0857, 91.0542	Phenylpropanoids
M48	9.57	DiosMetin 7-O-beta-D-glucuronide + CH_2_	C_23_H_22_O_12_	[M + H]^+^	491.1184	491.1193	1.83	315.0871, 300.0621	Flavonoids
M49	9.81	Oroxylin A + SO_3_	C_16_H_12_O_8_S	[M–H]^-^	363.0180	363.0186	1.57	283.0615, 268.0381	Flavonoids
M50	9.85	Wogonin + SO_3_	C_16_H_12_O_8_S	[M–H]^-^	363.0180	363.0184	1.17	283.0620, 268.0386	Flavonoids
M51	10.27	Wogonin + O	C_16_H_12_O_6_	[M + H]^+^	301.0707	301.0713	2.27	286.0496, 255.1266, 145.0174, 127.0064	Flavonoids
M52	10.76	β-Asarone-2CH_2_	C_10_H_12_O_3_	[M + H]^+^	181.0859	181.0864	2.71	139.0753, 135.0810, 107.0866	Phenylpropanoids
M53	11.09	Methyl eugenol-2CH_2_	C_10_H_12_O_2_	[M + H]^+^	165.0910	165.0912	1.11	137.0958, 121.0648, 119.0853, 107.0492, 91.0543	Phenylpropanoids
M54	11.63	β-Asarone-CH_2_	C_11_H_14_O_3_	[M + H]^+^	195.1016	195.1022	3.07	163.0390, 133.0315, 121.1020, 91.0529	Phenylpropanoids
M55	11.88	α-Asarone-2CH_2_	C_10_H_12_O_3_	[M + H]^+^	181.0859	181.0862	1.53	139.0732, 135.0797, 107.0847	Phenylpropanoids
M56	12.42	Oroxylin A + O	C_16_H_12_O_6_	[M + H]^+^	301.0707	301.0704	−0.87	283.0598, 268.0353, 240.0409, 227.0692	Flavonoids
M57	12.53	Wogonin+2H	C_16_H_14_O_5_	[M + H]^+^	287.0914	287.0920	2.23	183.0283, 175.0145, 168.0052, 159.0230, 131.0498	Flavonoids
M58	14.02	Methyl eugenol+2H	C_11_H_16_O_2_	[M + H]^+^	181.1223	181.1226	1.66	123.0801, 91.0548	Phenylpropanoids
M59	14.04	Dihydrooroxylin A + CH_2_	C_17_H_16_O_5_	[M + H]^+^	301.1071	301.1072	0.59	286.9927, 283.0514, 255.1261	Flavonoids
M60	19.96	Obtusifolin + CH_2_	C_17_H_14_O_5_	[M + H]^+^	299.0914	299.0914	0.16	283.0485, 269.0814, 250.9921, 226.0585	Anthraquinones

Note: M: metabolite component.

#### 3.5.2 Metabolites of flavonoid-related components analysis

**FIGURE 7:**
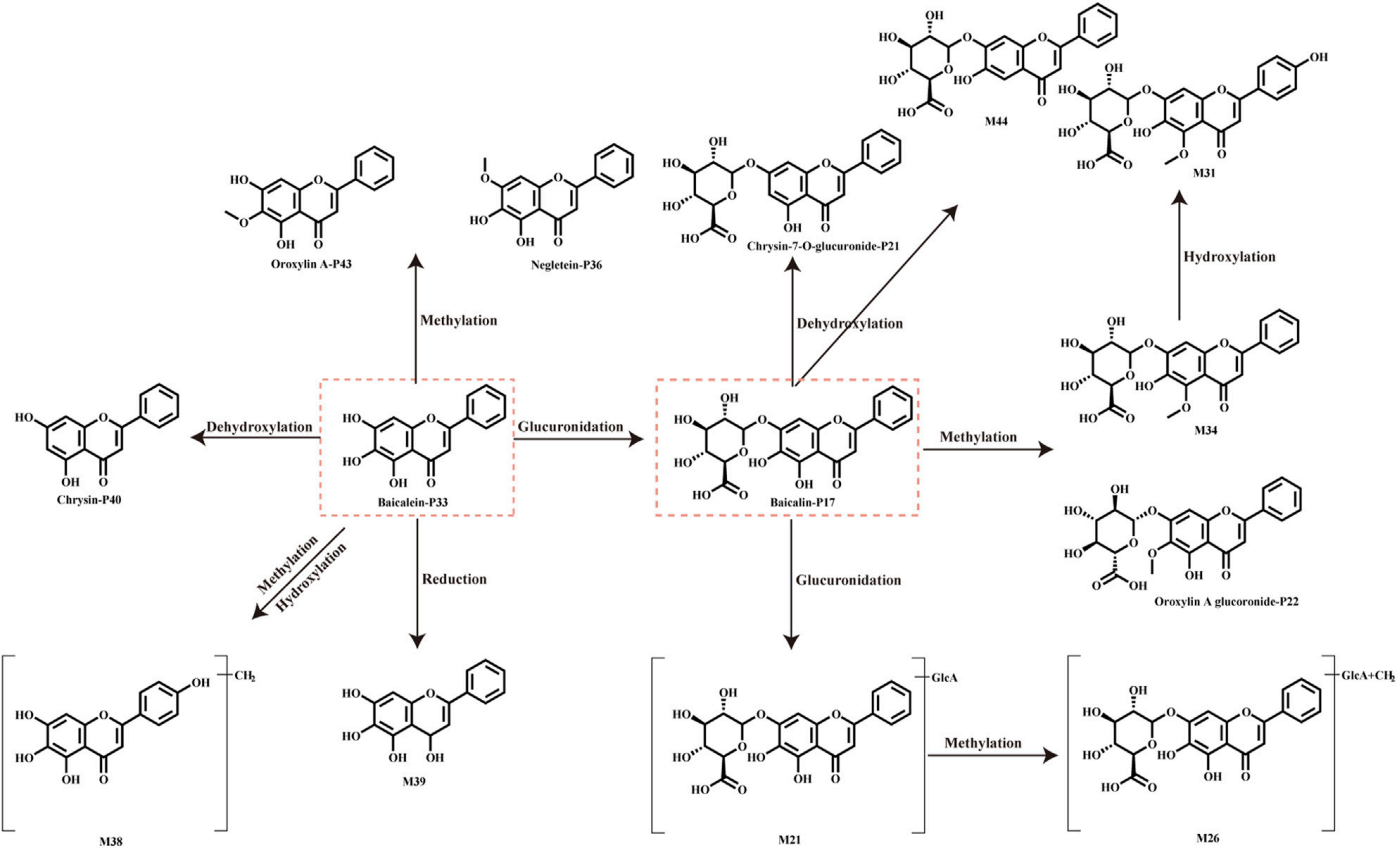
Main metabolic pathways of the flavonoids of baicalin and baicalein.

Flavonoids are the primary components of ASDZL *in vivo* after oral administration. Flavonoids demonstrate various pharmacological activities, including anti-inflammatory, antioxidant, and antibacterial effects, and are widely applied in active ingredient research and disease treatment (Wang et al., 2018). In this study, 57 flavonoids of prototypes and metabolites were identified in the biological samples. The primary metabolic pathways of flavonoids include methylation, demethylation, hydroxylation, hydroxylation, sulfation, and glucuronidation, for example, compound P17 (C_21_H_18_O_11,_ t_R_ = 7.34 min), for which the diagnostic ions *m/z* 269.0461 and *m/z* 169.0661 were observed in the negative MS^2^ spectrum due to the loss of glucuronic acid (GlcA, 176 Da) and RDA cleavage. The fragmentation ion of 251.0346 [M–H–C_6_H_8_O_6_–H_2_O]^-^ and *m/z* 223.0406 [M–H–C_6_H_8_O_6_–H_2_O–CO]^-^ was also detected. Thus, compound P17 was identified as baicalin. Subsequently, M34 (C_22_H_10_O_11,_ t_R_ = 7.34 min) in the negative MS^2^ spectrum yield the parent ion at *m/z* 459.0930 and the product ion at *m/z* 283.0620, which were both 14 Da more than P17. M34 was inferred as the methylation metabolite of P17. M21 (C27H26O17, t_R_ = 6.11 min) generated a quasi-molecular ion at *m/z* 621.1095, which was 176 Da higher than the parent ion of P17, and its product ion at *m/z* 269.0457 corresponded to P17 in the negative ion mode. Therefore, M21 was the glucuronidation metabolite of P17. Fragment ions at *m/z* 299.0507 and *m/z* 284.0351 indicated the loss of CH_3_ (−15 Da). On the basis of comparing parent ions and fragment ions, we speculated that P17 went through methylation and hydroxylation to produce M31. M44 (C_21_H_18_O_10,_ t_R_ = 9.12 min) yielded the parent ion at *m/z* 429.0841 and the product ion at *m/z* 253.0509, which were both 16 Da less than P17. Hence, M44 was inferred as the dihydroxylation metabolite of P17. The metabolic pathway of baicalin and baicalein are shown in Figure 7.

## 4 Conclusion

In this study, we integrated multiple strategies to successfully identify 243 components in ASDZL, with 61 components annotated by FBMN. In plasma samples, 50 prototypes and 60 metabolites were identified. The integrated analytical strategy used in this study contributes to the characterization of compounds in ASDZL, enabling further investigation of its pharmacological mechanisms.

## Data Availability

The raw mass spectrometry data generated in this study are available in the Figshare repository under accession number figshare.29975296. The data can be accessed at: https://doi.org/10.6084/m9.figshare.29975296.

## References

[B1] Chen T. Massias J. Bertrand S. Guitton Y. Le Bizec B. Dervilly G. (2024). Innovative molecular networking analysis of steroids and characterisation of the urinary steroidome. Sci. Data 11 (1), 818. 10.1038/s41597-024-03599-0 39048571 PMC11269682

[B2] Cho J. Hong E. B. Kim Y. A.-O. Song J. A.-O. Ju Y. H. Kim H. (2024). Baicalin and baicalein from *Scutellaria baicalensis* georgi alleviate aberrant neuronal suppression mediated by GABA from reactive astrocytes. CNS Neurosci. and Ther. 30 (5), e14740. 10.1111/cns.14740 38715318 PMC11076983

[B3] Dong X. Huang R. (2022). Ferulic acid: an extraordinarily neuroprotective phenolic acid with anti-depressive properties. Phytomedicine 105, 154355. 10.1016/j.phymed.2022.154355 35908520

[B4] Feng W. Ao H. Peng C. Yan D. (2019). Gut microbiota, a new frontier to understand traditional Chinese medicines. Pharmacol. Res. 142, 176–191. 10.1016/j.phrs.2019.02.024 30818043

[B5] Feng W. Yang Z. Liu Y. Chen R. Song Z. Pan G. (2023). Gut microbiota: a new target of traditional Chinese medicine for insomnia. Biomed. and Pharmacother. 160, 114344. 10.1016/j.biopha.2023.114344 36738504

[B6] Guo J. Shang Y. Yang X. Li J. He J. Gao X. (2022). An online stepwise background subtraction-based ultra-high pressure liquid chromatography quadrupole time of flight tandem mass spectrometry dynamic detection integrated with metabolic molecular network strategy for intelligent characterization of the absorbed chemical-fingerprint of QiangHuoShengShi decoction *in vivo* . J. Chromatogr. A 1675, 463172. 10.1016/j.chroma.2022.463172 35649309

[B7] Guo L.-T. Wang S.-Q. Su J. Xu L.-X. Ji Z.-Y. Zhang R.-Y. (2019). Baicalin ameliorates neuroinflammation-induced depressive-like behavior through inhibition of toll-like receptor 4 expression *via* the PI3K/AKT/FoxO1 pathway. J. Neuroinflammation 16 (1), 95. 10.1186/s12974-019-1474-8 31068207 PMC6507025

[B8] Han M. Xia H. Xia G. Wei X. Li J. Wu Y. (2025). Feature-based molecular networking (FBMN): an efficient booster for discovering novel natural products or metabolites. Acta Pharm. Sin. B 15 (4), 2283–2286. 10.1016/j.apsb.2025.02.019 40486865 PMC12138091

[B9] Heuckeroth S. Damiani T. Smirnov A. Mokshyna O. Brungs C. Korf A. (2024). Reproducible mass spectrometry data processing and compound annotation in MZmine 3. Nat. Protoc. 19 (9), 2597–2641. 10.1038/s41596-024-00996-y 38769143

[B10] Hong L.-l. Cui D.-x. Wang H.-d. Jing Q. Li X. Hu Y. (2025). Recent advances in traditional Chinese medicine metabolism: sample pre-treatment, MS-oriented analytical strategies and typical applications. TrAC Trends Anal. Chem. 189, 118269. 10.1016/j.trac.2025.118269

[B11] Jichao S. Xinmin H. Xianguo R. Dongqi Y. Rongyi Z. Shuang L. (2017). Saikosaponin A alleviates symptoms of attention deficit hyperactivity disorder through downregulation of DAT and enhancing BDNF expression in spontaneous hypertensive rats. Evidence-Based Complementary Altern. Med. 2017 (1), 2695903. 10.1155/2017/2695903 28293263 PMC5331296

[B12] Katajamaa M. Miettinen J. Orešič M. (2006). MZmine: toolbox for processing and visualization of mass spectrometry based molecular profile data. Bioinformatics 22 (5), 634–636. 10.1093/bioinformatics/btk039 16403790

[B13] Li L. Zhu N. Zhang L. Kuja-Halkola R. D’Onofrio B. M. Brikell I. (2024). ADHD pharmacotherapy and mortality in individuals with ADHD. Jama 331 (10), 850. 10.1001/jama.2024.0851 38470385 PMC10936112

[B14] Li X.-L. Guo Z.-F. Wen X.-D. Li M.-N. Yang H. (2023). A molecular networking-assisted automatic database screening strategy for comprehensive annotation of small molecules in complex matrices. J. Chromatogr. A 1710, 464417. 10.1016/j.chroma.2023.464417 37778098

[B15] Liu S. Ding P. Wu M. Zhu Z. Tao J. Wang J. (2023). Screening quality markers (Q-markers) of xiaoer chaige tuire oral liquid by *in vitro* sequential metabolism and *in vivo* biopharmaceutical analysis. Phytomedicine 116, 154844. 10.1016/j.phymed.2023.154844 37163902

[B16] Moreira J. Machado M. Dias-Teixeira M. Ferraz R. Delerue-Matos C. Grosso C. (2023). The neuroprotective effect of traditional Chinese medicinal plants—A critical review. Acta Pharm. Sin. B 13 (8), 3208–3237. 10.1016/j.apsb.2023.06.009 37655317 PMC10465969

[B17] Noh K. Kang Y. Nepal M. R. Jeong K. S. Oh D. G. Kang M. J. (2016). Role of intestinal microbiota in baicalin-induced drug interaction and its pharmacokinetics. Molecules 21, 337. 10.3390/molecules21030337 26978333 PMC6273104

[B18] Nothias L.-F. Petras D. Schmid R. Dührkop K. Rainer J. Sarvepalli A. (2020). Feature-based molecular networking in the GNPS analysis environment. Nat. Methods 17 (9), 905–908. 10.1038/s41592-020-0933-6 32839597 PMC7885687

[B19] Pakkir Shah A. K. Walter A. Ottosson F. Russo F. Navarro-Diaz M. Boldt J. (2024). Statistical analysis of feature-based molecular networking results from non-targeted metabolomics data. Nat. Protoc. 20 (1), 92–162. 10.1038/s41596-024-01046-3 39304763

[B20] Pan H. Yang W. Zhang Y. Yang M. Feng R. Wu W. (2015). An integrated strategy for the systematic characterization and discovery of new indole alkaloids from Uncaria rhynchophylla by UHPLC/DAD/LTQ-Orbitrap-MS. Anal. Bioanal. Chem. 407 (20), 6057–6070. 10.1007/s00216-015-8777-0 26055881

[B21] Pan L. Cho K.-S. Yi I. To C.-H. Chen D. F. Do C.-W. (2021). Baicalein, baicalin, and wogonin: protective effects against ischemia-induced neurodegeneration in the brain and retina. Oxidative Med. Cell. Longev. 2021 (1), 8377362. 10.1155/2021/8377362 34306315 PMC8263226

[B22] Pluskal T. Castillo S. Villar-Briones A. Orešič M. (2010). MZmine 2: modular framework for processing, visualizing, and analyzing mass spectrometry-based molecular profile data. BMC Bioinforma. 11 (1), 395. 10.1186/1471-2105-11-395 20650010 PMC2918584

[B23] Qu B. Liu Y. Shen A. Guo Z. Yu L. Liu D. (2023). Combining multidimensional chromatography-mass spectrometry and feature-based molecular networking methods for the systematic characterization of compounds in the supercritical fluid extract of Tripterygium wilfordii hook F. Analyst 148 (1), 61–73. 10.1039/d2an01471h 36441185

[B24] Ramanan M. Sinha S. Sudarshan K. Aidhen I. S. Doble M. (2016). Inhibition of the enzymes in the leukotriene and prostaglandin pathways in inflammation by 3-aryl isocoumarins. Eur. J. Med. Chem. 124, 428–434. 10.1016/j.ejmech.2016.08.066 27597418

[B25] Su Y. Tao L. Zhang X. Sheng X. Li Q. Fei W. (2023). Non-targeted characteristic filter analysis combined with *in silico* prediction strategies to identify the chemical components and *in vivo* metabolites of dalitong granules by UPLC-Q-TOF/MS/MS. J. Pharm. Biomed. Analysis 222, 115086. 10.1016/j.jpba.2022.115086 36219926

[B26] Sudarshan K. Yarlagadda S. Sengupta S. (2024). Recent advances in the synthesis of diarylheptanoids. Chem. – Asian J. 19 (15), e202400380. 10.1002/asia.202400380 38744677

[B27] van Dinteren S. Araya-Cloutier C. de Bruijn W. J. C. Vincken J.-P. (2021). A targeted prenylation analysis by a combination of IT-MS and HR-MS: identification of prenyl number, configuration, and position in different subclasses of (iso)flavonoids. Anal. Chim. Acta 1180, 338874. 10.1016/j.aca.2021.338874 34538332

[B28] Wang H. Lan Y. Luo L. Xiao Y. Meng X. Zeng Y. (2024). The scutellaria-coptis herb couple and its active small-molecule ingredient wogonoside alleviate cytokine storm by regulating the CD39/NLRP3/GSDMD signaling pathway. J. Ethnopharmacol. 329, 118155. 10.1016/j.jep.2024.118155 38593962

[B29] Wang M. Carver J. J. Phelan V. V. Sanchez L. M. Garg N. Peng Y. (2016). Sharing and community curation of mass spectrometry data with global natural products social molecular networking. Nat. Biotechnol. 34 (8), 828–837. 10.1038/nbt.3597 27504778 PMC5321674

[B30] Wang T.-y. Li Q. Bi K.-s. (2018). Bioactive flavonoids in medicinal plants: structure, activity and biological fate. Asian J. Pharm. Sci. 13 (1), 12–23. 10.1016/j.ajps.2017.08.004 32104374 PMC7032191

[B31] Wang X. Cao Y. Chen S. Lin J. Bian J. Huang D. (2021). Anti-inflammation activity of flavones and their structure–activity relationship. J. Agric. Food Chem. 69 (26), 7285–7302. 10.1021/acs.jafc.1c02015 34160206

[B32] Wang Y. Yang J. Fau - Li X. Li X Fau - Wang J. Wang J. (2012). The metabolism of baicalin in rat and the biological activities of the metabolites. Evidence-Based Complementary Altern. Med. 2012, 1741–1746. 10.1155/2012/404529 22654953 PMC3359663

[B33] Wei W. Han Q. Tian S. Wang Y. Zhang H. Wang H. (2022). Effective separation of α-asarone and β-asarone in TCM by covalent organic framework modified magnetic solid phase extraction. Microchem. J. 175, 107015. 10.1016/j.microc.2021.107015

[B34] Xu Q. Liu J. Wang X. jiao Y. Zhang Y. Shang X. (2025). Characterization, target isolation of triterpenes in the anti-inflammatory fraction of Salvia rosmarinus *via* UPLC-orbitrap MS/MS coupled with GNPS. J. Agric. Food Chem. 73 (18), 10985–10997. 10.1021/acs.jafc.4c12650 40266574

[B35] Yang C. Yin X. Dong X. Zhang X. You L. Wang W. (2017). Determination of the phytochemical composition of jingning Fang and the *in vivo* pharmacokinetics of its metabolites in rat plasma by UPLC–MS/MS. J. Chromatogr. B 1067, 71–88. 10.1016/j.jchromb.2017.09.019 29017076

[B36] Yaqun L. Haixia Y. Yuchen S. Mingxin Z. Manqi L. Yunlong T. (2022). An shen ding zhi ling ameliorates the symptoms of attention deficit hyperactivity disorder *via* modulating brain-derived neurotrophic factor-related signaling pathways. Evidence-Based Complementary Altern. Med. 2022, 1–13. 10.1155/2022/5471586 35911131 PMC9334057

[B37] Yuan H. Ni X. Zheng M. Han X. Song Y. Yu M. (2019). Effect of catalpol on behavior and neurodevelopment in an ADHD rat model. Biomed. and Pharmacother. 118, 109033. 10.1016/j.biopha.2019.109033 31545235

[B38] Zhang J. Tan B. Wu H. Han T. Fang D. Cai H. (2025). Scutellaria baicalensis extracts restrict intestinal epithelial cell ferroptosis by regulating lipid peroxidation and GPX4/ACSL4 in colitis. Phytomedicine 141, 156708. 10.1016/j.phymed.2025.156708 40220415

[B39] Zhang J.-j. Mao m. Shao M.-m. Wang M.-c. (2024a). Therapeutic potential of natural flavonoids in pulmonary arterial hypertension: a review. Phytomedicine 128, 155535. 10.1016/j.phymed.2024.155535 38537442

[B40] Zhang L. Yong Y.-Y. Deng L. Wang J. Law B. Y.-K. Hu M.-L. (2023). Therapeutic potential of polygala saponins in neurological diseases. Phytomedicine 108, 154483. 10.1016/j.phymed.2022.154483 36260972

[B41] Zhang Y. Liao J. Le W. Zhang W. Wu G. (2024b). In-Depth analysis of molecular network based on liquid chromatography coupled with tandem mass spectrometry in natural products: importance of redundant nodes discovery. Anal. Chem. 96 (40), 15888–15897. 10.1021/acs.analchem.4c02230 39311834

[B42] Zhao P. Qiu J. Pan C. Tang Y. Chen M. Song H. (2023). Potential roles and molecular mechanisms of bioactive ingredients in curcumae rhizoma against breast cancer. Phytomedicine 114, 154810. 10.1016/j.phymed.2023.154810 37075623

[B43] Zhou R. Wang J. Han X. Ma B. Yuan H. Song Y. (2019). Baicalin regulates the dopamine system to control the core symptoms of ADHD. Mol. Brain 12 (1), 11. 10.1186/s13041-019-0428-5 30736828 PMC6368814

[B44] Zhu H. He L. Wu W. Duan H. Chen J. Xiao Q. (2023). A compounds annotation strategy using targeted molecular networking for offline two-dimensional liquid chromatography-mass spectrometry analysis: Yupingfeng as a case study. J. Chromatogr. A 1702, 464045. 10.1016/j.chroma.2023.464045 37236139

