# Supplementary material for: UHPLC-HRMS analysis combined with feature-based molecular networking methods for systematic identification of chemicals in AnShenDingZhiLing and its absorbed metabolites
Source: Front Chem. 2025 Sep 3;13:1647159. doi: 10.3389/fchem.2025.1647159 (PMC12441513; doi:10.3389/fchem.2025.1647159)
Supplement: Supplementary file 1 [file DataSheet1.docx]

Supplementary Material

# Supplementary Methods, Figures and Tables

## The detailed chemical and herbal information

Rhynchophylline (batch number: 21122302) and isorhynchophylline (batch number: 20022101) were obtained from Pufei De Biotech Co., Ltd. (Chengdu, China). Baicalin (batch number: RM0523FA14) and saikosaponin A (batch number: W25J6K1919) were purchased from Shanghai Yuanye Bio-technology Co., Ltd. (Shanghai, China). Catalpol (batch number: RFS-Z00511803015), salidroside (batch number: RDD-H04002404015), ferulic Acid (batch number: RFS-A00211812016), polygalaxanthone III (batch number: RDD-Y05511712016), scutellarin (batch number: RDD-Y01202401005), forsythoside A (batch number: RDD-L01211812016), verbascoside (batch number: M-011-191014), (3-sinapoyl) fructofuranosyl-(6-sinapoyl) glucopyranoside (batch number: RDD-F01302210010), scutellarein (batch number: RDD-G04701907023), quercetin (batch number: RDD-H00902211023), emodin (batch number: RFS-D02902203030), chrysophanic acid (batch number: RFS-D01711811012) and wogonoside (batch number: RDD-H01911809030) were purchased from Herbpurify Co., Ltd. (Chengdu, China). The purity of above compounds was more than 98%. *Scutellaria baicalensis* Georgi (batch number: 2240501), *Bupleurum chinense* DC. (batch number: 2240501), *Angelica sinensis* (Oliv.) Diels (batch number: 240501), *Rehmannia glutinosa* (Gaertn.) Libosch. ex DC. (batch number: 240501), *Uncaria rhynchophylla* (Miq.), Miq. ex Havil. (batch number: 240102), *Senna obtusifolia* (L.) H.S.Irwin & Barneby (batch number: 231001), *Forsythia suspensa* (Thunb.) Vahl (batch number: 221206), *Acorus calamus* var. angustatus Besser (batch number: 240401), *Alpinia oxyphylla* Miq. (batch number: 231001), *Curcuma aromatica* Salisb. (batch number: 2301071), *Polygala tenuifolia* Willd. (batch number: 231101), *Bambusa textilis* McClure (batch number: 231001), were purchased from Anhui Jingwan Pharmacy Chain Ltd. All listed Chinese herbal medicines conform to the Chinese Pharmacopoeia 2020 edition.

## Supplementary Figures


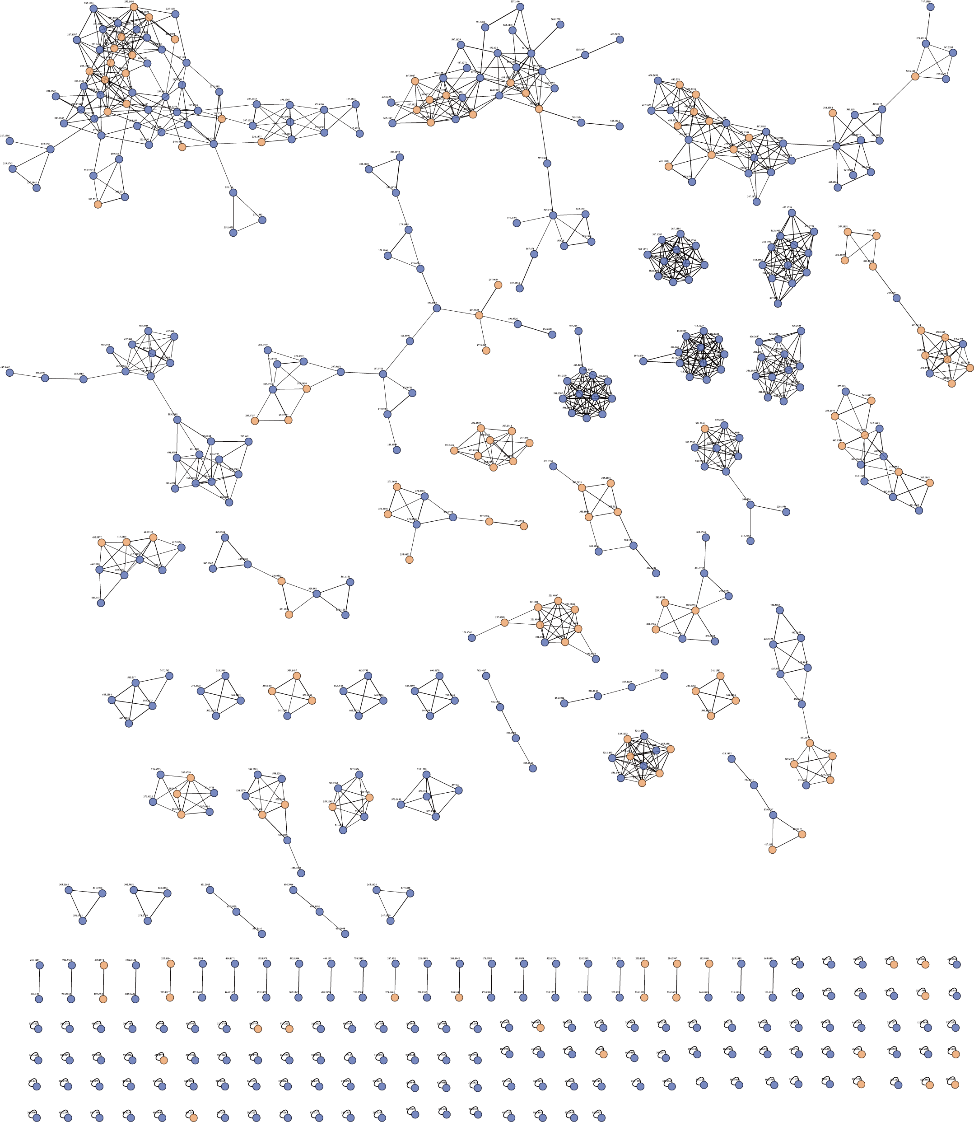


**Supplementary Figure S1.** Feature-based molecular networking (FBMN) at positive ion mode of ASDZL (Red and blue nodes represented GNPS Library Database annotated and non-annotated compounds). FBMN task ID: <https://gnps.ucsd.edu/ProteoSAFe/status.jsp?task=bce7a4e5175b45068a1f6b93a345f46a>

**
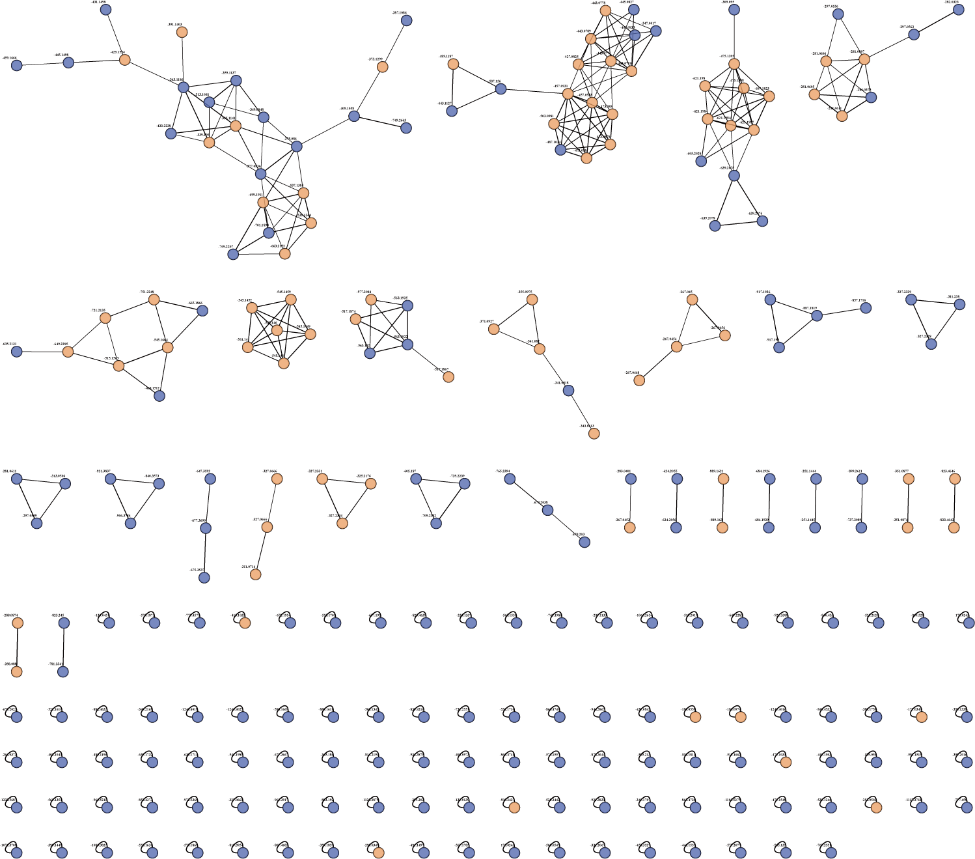
**

**Supplementary Figure S2.** Feature-based molecular networking (FBMN) at negative ion mode of ASDZL (Red and blue nodes represented GNPS Library Database annotated and non-annotated compounds). FBMN task ID: <https://gnps.ucsd.edu/ProteoSAFe/status.jsp?task=6d5747bf8eae409aa54836e5851dc1c2>


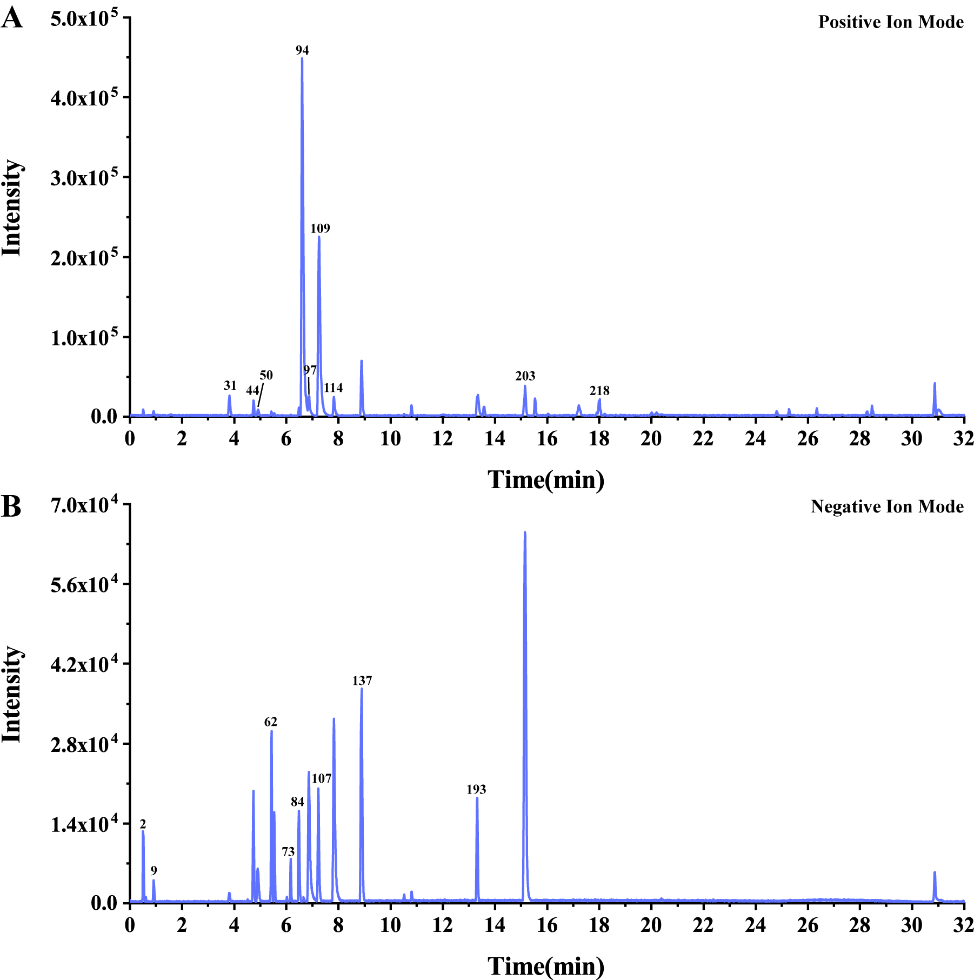


**Supplementary Figure S3.** Base peak chromatogram (BPC) of reference standards in UHPLC-QTOF MS positive (A) and negative (B) ion modes.


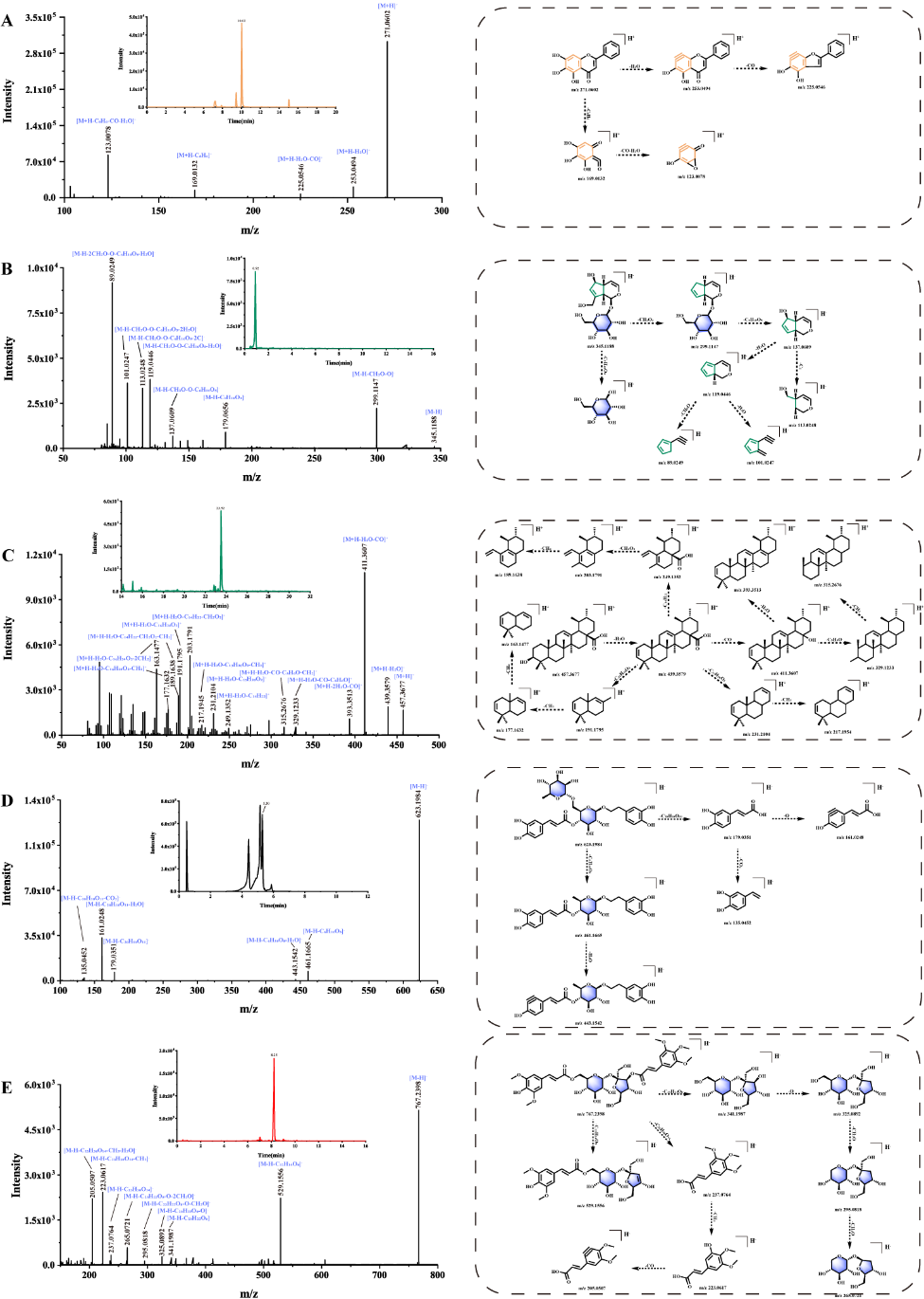


**Supplementary Figure S4.** Fragmentation routes of main components of ASDZL. (A) Baicalein; (B) Aucubin; (C) Ursolic Acid; (D) Forsythoside A; (E) Tenuifoliside C.


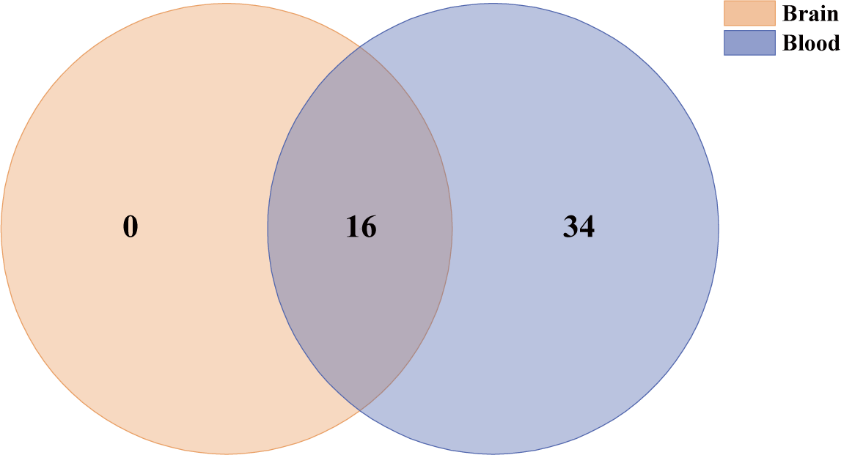


**Supplementary Figure S5.** The Venn diagram of 50 prototypes from bio-samples


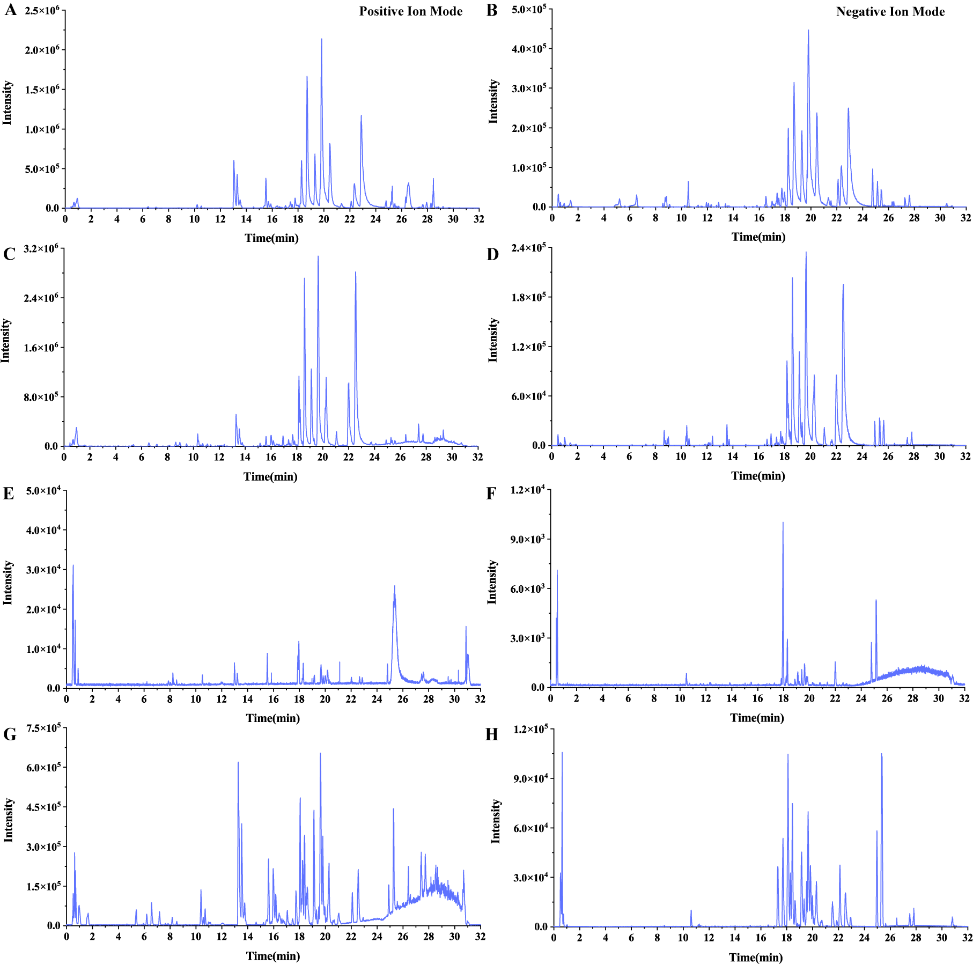


**Supplementary Figure S6.** The base peak chromatogram (BPC) of the bio-samples from the rats in control group and the administration group both in positive mode (A: plasma- blank, C: plasma-ASDZL, E; cerebrum-blank, G: cerebrum-ASDZL) and in negative mode (B: plasma- blank, D: plasma-ASDZL, F; cerebrum-blank, H: cerebrum-ASDZL) by UHPLC-QTOF-

## Supplementary Tables

Table S1 61 constituents annotated by Feature-based molecular networking.

| Identification | Sources | Types | Identification | Sources | Types |
| --- | --- | --- | --- | --- | --- |
| Oleanolic Acid | Unc | Terpenoids | Chrysin | Scu | Flavonoids |
| Nootkatone | Alp | Terpenoids | Dihydrooroxylin A | Scu | Flavonoids |
| Jaeschkeanadiol | Alp | Terpenoids | Rhynchophylline | Unc | Alkaloids |
| Beta-Caryophyllene oxide | Aco | Terpenoids | Isorhynchophylline | Unc | Alkaloids |
| Zedoarondiol | Cur | Terpenoids | Corynoxine | Unc | Alkaloids |
| Chrysin-7-O-glucuronide | Scu | Flavonoids | Corynoxeine | Unc | Alkaloids |
| Baicalin | Scu | Flavonoids | Hirsuteine | Unc | Alkaloids |
| Hispidulin 7-glucuronide | Scu | Flavonoids | Geissoschizine methyl ether | Unc | Alkaloids |
| Prunin | Sen | Flavonoids | Yohimbine | Unc | Alkaloids |
| Tricin 5-glucoside | Sen | Flavonoids | Hirsutine | Unc | Alkaloids |
| Oroxylin A glucoronide | Scu | Flavonoids | Aurantio-obtusin | Sen | Anthraquinones |
| Calceolarioside B | For | Phenylethanoid glycosides | Oroxylin A | Scu | Flavonoids |
| Rutin | For | Flavonoids | Scutellarein 4'-methyl ether | Scu | Flavonoids |
| Feruloyltyramine | Aco | Alkaloids | Wogonin | Scu | Flavonoids |
| Forsythoside A | For | Phenylethanoid glycosides | Matairesinol | For | Phenylpropanoids |
| Cadambine | Unc | Alkaloids | Strictosamide | Unc | Alkaloids |
| Calceolarioside C | For | Phenylethanoid glycosides | Emodin | Sen | Anthraquinones |
| Isorhamnetin | Bup | Flavonoids | Darendoside A | For | Phenylethanoid glycosides |
| Obtusifolin | Sen | Anthraquinones | Oroxindin | Scu | Flavonoids |
| Physcione | Sen | Anthraquinones | 3,4,5-Trihydroxy-6-[5-hydroxy-2-(4-hydroxy-3-methoxyphenyl)-3-methoxy-4-oxochromen-7-yl] oxyoxane-2-carboxylic acid | **/** | Flavonoids |
| 2-(3-Methoxy-4-hydroxyphenyl)-5-(3,4-dimethoxyphenyl)-3,4-dimethyltetrahydrofuran | For | Phenylpropanoids | Calceolarioside A | For | Phenylethanoid glycosides |
| (+)-Grandisin | For | Phenylpropanoids | [(2R,3R,4S,5R,6R)-6-[2-(3,4-dihydroxyphenyl) ethoxy]-3,5-dihydroxy-4-[(3R,4R,5R,6S)-3,4,5-trihydroxy-6-methyloxan-2-yl] oxyoxan-2-yl] methyl (E)-3-(3,4-dihydroxyphenyl) prop-2-enoate | For | Phenylethanoid glycosides |
| Skullcapflavone II | Scu | Flavonoids | Sibiricose A5 | Pol | Oligosaccharides |
| 5,2',6'-Trihydroxy-6,7,8-trimethoxyflavone | **/** | Flavonoids | (3-sinapoyl) fructofuranosyl-(6-sinapoyl) glucopyranoside | Pol | Oligosaccharides |
| Ursolic Acid | Unc | Terpenoids | Sibiricose A1 | Pol | Oligosaccharides |
| 3,4,5-Trimethoxycinnamic acid | Ang | Organic acids | Isoschaftoside | Scu | Flavonoids |
| 5,7-Dihydroxy-2-phenyl-6-[3,4,5-trihydroxy-6-(hydroxymethyl) oxan-2-YL]-8-(3,4,5-trihydroxyoxan-2-YL) chromen-4-one | **/** | Flavonoids | Phillyrin | For | Phenylpropanoids |
| 5,7-Dihydroxy-2-phenyl-6,8-bis[3,4,5-trihydroxy-6-(hydroxymethyl) oxan-2-yl] chromen-4-one | **/** | Flavonoids | Pinoresinol 4-O-glucoside | For | Phenylpropanoids |
| Baicalein | Scu | Flavonoids | Matairesinoside | For | Phenylpropanoids |
| Saikosaponin B2 | Bup | Terpenoids | Saikosaponin A | Bup | Terpenoids |
| Cryptochlorogenic acid | Ang | Phenolic acids |  |  |  |

Unc: *Uncaria rhynchophylla* (Miq.) Miq. ex Havil.; Alp: *Alpinia oxyphylla* Miq.; Aco: *Acorus calamus* var. angustatus Besser;

Cur: *Curcuma aromatica* Salisb.; Scu: *Scutellaria baicalensis* Georgi; Sen: *Senna obtusifolia* (L.) H.S.Irwin & Barneby;

For: *Forsythia suspensa* (Thunb.) Vahl; Bup: *Bupleurum chinense* DC.; Pol: *Polygala tenuifolia* Willd;

Ang: *Angelica sinensis* (Oliv.) Diels; **/**: Unknown

Table S2 Summary of diagnostic ions and neutral losses from different structure types.

| Typical components | Structure types | Subtypes | Mode | Diagnostic ions | Neutral losses |
| --- | --- | --- | --- | --- | --- |
| Baicalin | Flavonoids | Flavone glycosides | NEG | 269.0551,153.0715 | C_6_H_8_O_6_, H_2_O, CO, O |
| Scutellarein | Flavonoids | Flavonoids | POS | 169.0130 | H_2_O, CO, O |
| Jaeschkeanadiol | Terpenoids | Sesquiterpenes | POS | / | H_2_O, CH_2_ |
| Catalpol | Terpenoids | monoterpenes | NEG | 179.0572,89.0259 | C_6_H_10_O_5_, H_2_O, CH_2_O, O |
| Saikosaponin A | Terpenoids | Triterpenoid saponins | NEG | 617.4052 | C_6_H_10_O_5_ |
| Ursolic Acid | Terpenoids | Triterpenes | POS | 411.3607,249.1352,203.1791,191.1795 | H_2_O, CO, CH_2_, CO_2_ |
| Chrysophanol | Anthraquinones | Anthraquinones | POS | 237.0540,227.0702 | CO, H_2_O |
| Isorhynchophylline | Alkaloids | Indole alkaloids | POS | 353.1859,160.0756,144.0809 | CH_3_OH |
| Salidroside | Terpenoids | Phenylpropanoids | NEG | 179.0356,137.0248 | C_6_H_10_O_5_, H_2_O, CH_2_O |
| Forsythoside A | Phenylethanoid glycosides | Phenylethanoid glycosides | NEG | 461.1665,173.0351 | C_6_H_10_O_5_, H_2_O, CO_2_ |
| (3-sinapoyl) fructofuranosyl-(6-sinapoyl) glucopyranoside | Oligosaccharide | Oligosaccharide | NEG | 547.1675,223.0612,205.0525 | C_11_H_10_O_4_, C_6_H_10_O_5_, H_2_O |
| polygalaxanthone III | Xanthones | Xanthones | POS | 437.1082,275.0552 | C_5_H_8_O_4_, C_6_H_10_O_5_, H_2_O, CH_2_O |
| Ferulic Acid | Phenolic acids | Phenolic acids | POS | 177.0552 | H_2_O, CO |

**Table S3** Characteristics of chemical components in ASDZL by UHPLC-­QTOF-MS.

| NO. | RT  (min) | Identification | Formular | Selected ion | Theoretical Mass (Da) | Measured Mass (Da) | Error  (ppm) | MS/MS fragments | Types |
| --- | --- | --- | --- | --- | --- | --- | --- | --- | --- |
| 1 | 0.51 | Quinic acid | C_7_H_12_O_6_ | [M-H]^-^ | 191.0561 | 191.0557 | -2.01 | 173.0456,127.0408,111.0103,93.0354 | Organic acids |
| 2 | 0.62 | Catalpol* | C_15_H_22_O_10_ | [M-H]^-^ | 361.1140 | 361.1139 | -0.22 | 315.1100,179.0572,161.0455,153.0563,  135.0461,119.0356,101.0252,89.0259 | Terpenoids |
| 3 | 0.67 | Vanillic acid | C_8_H_8_O_4_ | [M-H]^-^ | 167.0350 | 167.0347 | -1.69 | 123.0457,108.0234 | Phenolic acids |
| 4 | 0.74 | 8-Epiloganic acid | C_16_H_24_O_10_ | [M-H]^-^ | 375.1297 | 375.1299 | 0.63 | 213.0790,179.0550,169.0879,151.0772,  125.0627,89.0249 | Terpenoids |
| 5 | 0.81 | Chlorogenic Acid | C_16_H_18_O_9_ | [M-H]^-^ | 353.0878 | 353.0879 | 0.32 | 191.0581,179.0361,135.0466 | Phenolic acids |
| 6 | 0.81 | Cistanoside F | C_21_H_28_O_13_ | [M-H]^-^ | 487.1457 | 487.1450 | -1.54 | 203.0357,179.0353,161.0246,135.0448 | Phenylethanoid glycosides |
| 7 | 0.83 | Forsythoside E | C_20_H_30_O_12_ | [M-H]^-^ | 461.1665 | 461.1666 | 0.39 | 317.1100,205.0740,163.0625,143.0362,  135.0485 | Phenylethanoid glycosides |
| 8 | 0.85 | Protocatechuic acid | C_7_H_6_O_4_ | [M-H]^-^ | 153.0193 | 153.0192 | -0.65 | 135.0460,123.0457,109.0314,91.0192 | Phenolic acids |
| 9 | 0.91 | Salidroside* | C_14_H_20_O_7_ | [M-H]^-^ | 299.1136 | 299.1134 | -0.72 | 179.0356,137.0248,119.0497,89.0251 | Phenylethanoid glycosides |
| 10 | 0.92 | Aucubin | C_15_H_22_O_9_ | [M-H]^-^ | 345.1191 | 345.1188 | -0.92 | 299.1147,179.0565,137.0609,119.0446,  113.0248,101.0247,89.0249 | Terpenoids |
| 11 | 1.04 | Darendoside A | C_19_H_28_O_11_ | [M-H]^-^ | 431.1559 | 431.1555 | -0.83 | 299.1143,191.0550,179.0546,149.0455,  137.0613,119.0347 | Phenylethanoid glycosides |
| 12 | 1.14 | Catechin | C_15_H_14_O_6_ | [M+H]^+^ | 291.0863 | 291.0875 | 4.11 | 165.0535,161.0593,147.0440,139.0392,  123.0441 | Flavonoids |
| 13 | 1.18 | Cryptochlorogenic acid | C_16_H_18_O_9_ | [M-H]^-^ | 353.0878 | 353.0880 | 0.50 | 191.0579,179.0355,173.0459,135.0453 | Phenolic acids |
| 14 | 1.22 | 3,4-Dihydroxybenzaldehyde | C_7_H_6_O_3_ | [M-H]^-^ | 137.0244 | 137.0244 | 0.08 | 136.0175,119.0141,109.0299,108.0224,  93.0353 | Aromatic aldehydes |
| 15 | 1.27 | Sibiricose A5 | C_22_H_30_O_14_ | [M-H]^-^ | 517.1563 | 517.1564 | 0.32 | 193.0510,175.0409 | Oligosaccharides |
| 16 | 1.39 | 3-O-Feruloylquinic acid | C_17_H_20_O_9_ | [M+H]^+^ | 369.1180 | 369.1181 | 0.36 | 207.0694,175.0395,147.0441,119.0494,  91.0543 | Phenolic acids |
| 17 | 1.40 | Sibiricose A6 | C_23_H_32_O_15_ | [M-H]^-^ | 547.1668 | 547.1666 | -0.37 | 223.0614,205.0506,190.0270 | Oligosaccharides |
| 18 | 1.41 | Quercetin 4′-O-glucoside | C_21_H_20_O_12_ | [M-H]^-^ | 463.0882 | 463.0875 | -1.54 | 301.0352,283.0250,175.0034,151.0036 | Flavonoids |
| 19 | 1.50 | Esculetin | C_9_H_6_O_4_ | [M-H]^-^ | 177.0193 | 177.0192 | -0.97 | 149.0244,133.0299,105.0350 | Phenylpropanoids |
| 20 | 1.56 | Caffeic acid | C_9_H_8_O_4_ | [M-H]^-^ | 179.0350 | 179.0350 | 0.32 | 135.0523,117.0349,107.0506,89.0399 | Phenolic acids |
| 21 | 1.60 | Syringic acid | C_9_H_10_O_5_ | [M+H]^+^ | 199.0601 | 199.0599 | -1.12 | 181.0500,155.0691,139.0378,125.0234,  95.0491 | Phenolic acids |
| 22 | 1.61 | Protocatechuic acid methyl ester | C_8_H_8_O_4_ | [M-H]^-^ | 167.0350 | 167.0346 | -2.47 | 152.0111,123.0455,108.0210 | Phenolic acids |
| 23 | 1.80 | L-Epicatechin | C_15_H_14_O_6_ | [M+H]^+^ | 291.0863 | 291.0863 | 0.07 | 165.0566,161.0598,147.0440,139.0392,  123.0443 | Flavonoids |
| 24 | 2.35 | Isovanillin | C_8_H_8_O_3_ | [M+H]^+^ | 153.0546 | 153.0547 | 0.37 | 125.0596,110.0358,93.0335 | Aromatic aldehydes |
| 25 | 2.44 | Sibiricose A1 | C_23_H_32_O_15_ | [M-H]^-^ | 547.1668 | 547.1661 | -1.28 | 265.0712,223.0610,205.0510 | Oligosaccharides |
| 26 | 2.45 | Isoeugenol | C_10_H_12_O_2_ | [M+H]^+^ | 165.0910 | 165.0909 | -0.75 | 147.0444,119.0489,91.0543 | Phenylpropanoids |
| 27 | 2.50 | Eugenol | C_10_H_12_O_2_ | [M+H]^+^ | 165.0910 | 165.0908 | -1.55 | 147.0442,119.0489,91.0544 | Phenylpropanoids |
| 28 | 2.55 | Vanillin | C_8_H_8_O_3_ | [M+H]^+^ | 153.0546 | 153.0544 | -1.15 | 125.0596,110.0361,93.0335 | Aromatic aldehydes |
| 29 | 2.63 | p-Coumaric acid | C_9_H_8_O_3_ | [M-H]^-^ | 163.0401 | 163.0396 | -3.11 | 119.0507,93.0348 | Phenolic acids |
| 30 | 3.27 | Scopoletin | C_10_H_8_O_4_ | [M+H]^+^ | 193.0495 | 193.0496 | 0.52 | 178.0256,165.0542,150.0306,133.0283 | Phenylpropanoids |
| 31 | 3.40 | Ferulic acid* | C_10_H_10_O_4_ | [M+H]^+^ | 195.0652 | 195.0649 | -1.47 | 177.0552,163.0390,149.0594,145.0285,  117.0337,89.0389 | Phenolic acids |
| 32 | 3.57 | Purpureaside C | C_35_H_46_O_20_ | [M-H]^-^ | 785.2510 | 785.2501 | -1.16 | 623.2179,161.0248 | Phenylethanoid glycosides |
| 33 | 3.59 | Lancerin | C_19_H_18_O_10_ | [M+H]^+^ | 407.0973 | 407.0970 | -0.68 | 371.0751,353.0643,335.0565,287.0547,  257.0439,241.0508 | Xanthones |
| 34 | 3.64 | Sibiricaxanthone B | C_24_H_26_O_14_ | [M-H]^-^ | 537.1250 | 537.1245 | -0.95 | 405.0835,387.0723,315.0517,297.0405,  285.0406,267.0306 | Xanthones |
| 35 | 3.68 | Isoschaftoside | C_26_H_28_O_14_ | [M-H]^-^ | 563.1406 | 563.1403 | -0.58 | 473.1087,443.0978,383.0775,353.0663 | Flavonoids |
| 36 | 3.74 | Rengynic acid | C_8_H_14_O_4_ | [M-H]^-^ | 173.0819 | 173.0817 | -1.58 | 129.0923,111.0817 | Organic acids |
| 37 | 4.08 | 5,7-Dihydroxy-2-phenyl-6,8-bis[3,4,5-trihydroxy-6-(hydroxymethyl) oxan-2-yl] chromen-4-one | C_27_H_30_O_14_ | [M-H]^-^ | 577.1563 | 577.1554 | -1.59 | 487.1233,457.1139,367.0821,337.0719 | Flavonoids |
| 38 | 4.21 | 8-Hydroxypinoresinol 4'-glucoside | C_26_H_32_O_12_ | [M-H]^-^ | 535.1821 | 535.1813 | -1.42 | 373.1297,343.1189,313.1086,181.0506,  151.0397 | Phenylpropanoids |
| 39 | 4.34 | Polygalaxanthone XI | C_25_H_28_O_15_ | [M+H]^+^ | 569.1501 | 569.1500 | -0.11 | 437.1082,419.0978,341.0654,317.0649,  287.0544,275.0552 | Xanthones |
| 40 | 4.40 | Calceolarioside A | C_23_H_26_O_11_ | [M-H]^-^ | 477.1402 | 477.1390 | -2.59 | 179.0354,161.0251,135.0459 | Phenylethanoid glycosides |
| 41 | 4.50 | Zedoalactone C | C_15_H_22_O_4_ | [M+H]^+^ | 267.1591 | 267.1594 | 1.14 | 249.1486,231.1379,213.1282,185.1324,  175.0758,161.1321,147.0799,133.1005 | Terpenoids |
| 42 | 4.54 | Rehmapicroside | C_16_H_26_O_8_ | [M-H]^-^ | 345.1555 | 345.1549 | -1.62 | 179.0549,165.0924,119.0349,101.0245 | Terpenoids |
| 43 | 4.55 | Rutin | C_27_H_30_O_16_ | [M+H]^+^ | 611.1607 | 611.1611 | 0.78 | 465.1021,303.0611 | Flavonoids |
| 44 | 4.59 | Polygalaxanthone III* | C_25_H_28_O_15_ | [M+H]^+^ | 569.1501 | 569.1497 | -0.67 | 437.1089,419.0985,401.0864,383.0755,  341.0653,317.0663,287.0553,275.0552 | Xanthones |
| 45 | 4.64 | Jionoside A1 | C_36_H_48_O_20_ | [M-H]^-^ | 799.2666 | 799.2664 | -0.23 | 753.2195,623.2192,429.2361 | Phenylethanoid glycosides |
| 46 | 4.67 | Calceolarioside C | C_28_H_34_O_15_ | [M+NH4]^+^ | 628.2236 | 628.2232 | -0.65 | 479.1558,457.1337,325.0932,163.0387 | Phenylethanoid glycosides |
| 47 | 4.75 | Chrysin 6-C-arabinoside 8-C-glucoside | C_26_H_28_O_13_ | [M+H]^+^ | 549.1603 | 549.1603 | 0.06 | 531.1551,495.1320,465.1210,417.0986,  387.0869,363.0398 | Flavonoids |
| 48 | 4.78 | Coniferaldehyde | C_10_H_10_O_3_ | [M+H]^+^ | 179.0703 | 179.0709 | 3.66 | 161.0603,133.0649,105.0697 | Phenylpropanoids |
| 49 | 4.80 | Cadambine | C_27_H_32_N_2_O_10_ | [M+H]^+^ | 545.2130 | 545.2128 | -0.25 | 383.1606,365.1493,227.1181 | Alkaloids |
| 50 | 4.82 | Scutellarin* | C_21_H_18_O_12_ | [M+H]^+^ | 463.0871 | 463.0866 | -1.04 | 287.0566 | Flavonoids |
| 51 | 4.89 | Torachrysone | C_14_H_14_O_4_ | [M+H]^+^ | 247.0965 | 247.0964 | -0.41 | 229.1195,205.0864,177.0912,161.0595 | Naphthopyrones |
| 52 | 4.95 | Xi-3-Hydroxy-5-phenylpentanoic acid O-beta-D-Glucopyranoside | C_17_H_24_O_8_ | [M+NH4]^+^ | 374.1809 | 374.1819 | 2.64 | 195.1003,177.0912,159.0800,131.0851,  117.0696,91.0542 | Organic acids |
| 53 | 4.97 | Tenuifoliside B | C_30_H_36_O_17_ | [M-H]^-^ | 667.1880 | 667.1870 | -1.44 | 461.1300,205.0508,137.0245 | Oligosaccharides |
| 54 | 5.01 | 4-Hydroxybenzoic acid | C_7_H_6_O_3_ | [M-H]^-^ | 137.0244 | 137.0236 | -5.84 | 93.0356 | Phenolic acids |
| 55 | 5.10 | Umbelliferone 7-O-Rutinoside | C_21_H_26_O_12_ | [M+H]^+^ | 471.1497 | 471.1497 | -0.01 | 325.0916,163.0393,145.0286,129.0546 | Phenylpropanoids |
| 56 | 5.10 | Forsythoside G | C_35_H_46_O_19_ | [M+NH4]^+^ | 788.2972 | 788.2955 | -2.17 | 617.2069,471.1487,325.0909,309.0971,  293.1263,239.0914,181.0477,163.0390,  147.0646,129.0549 | Phenylethanoid glycosides |
| 57 | 5.15 | [(2R,3R,4S,5R,6R)-6-[2-(3,4-dihydroxyphenyl) ethoxy]-3,5-dihydroxy-4-[(3R,4R,5R,6S)-3,4,5-trihydroxy-6-methyloxan-2-yl] oxyoxan-2-yl] methyl (E)-3-(3,4-dihydroxyphenyl) prop-2-enoate | C_29_H_36_O_15_ | [M-H]^-^ | 623.1981 | 623.1986 | 0.66 | 461.1669,443.1556,179.0353,161.0252,  135.0453 | Phenylethanoid glycosides |
| 58 | 5.19 | Emodin-1-O-β-gentiobioside | C_27_H_30_O_15_ | [M+H]^+^ | 597.1814 | 597.1814 | 0.05 | 435.1295,273.0770 | Anthraquinones |
| 59 | 5.22 | Tricin 5-glucoside | C_23_H_24_O_12_ | [M-H]^-^ | 491.1195 | 491.1190 | -1.08 | 329.0666,314.0434,299.0190 | Flavonoids |
| 60 | 5.25 | Umbelliferone | C_9_H_6_O_3_ | [M+H]^+^ | 163.0390 | 163.0391 | 0.67 | 145.0302,135.0468,117.0367,89.0444 | Phenylpropanoids |
| 61 | 5.25 | Calceolarioside B | C_23_H_26_O_11_ | [M+H]^+^ | 479.1548 | 479.1544 | -0.91 | 461.1425,443.1351,325.0915,163.0408,  145.0288,135.0444,117.0335 | Phenylethanoid glycosides |
| 62 | 5.30 | Forsythoside A* | C_29_H_36_O_15_ | [M-H]^-^ | 623.1981 | 623.1984 | 0.42 | 461.1665,443.1543,179.0351,161.0248,  135.0452 | Phenylethanoid glycosides |
| 63 | 5.32 | Zedoalactone A | C_15_H_22_O_4_ | [M+H]^+^ | 267.1591 | 267.1587 | -1.64 | 249.1473,231.1379,213.1267,185.1322 | Terpenoids |
| 64 | 5.39 | 5,7-Dihydroxy-2-phenyl-6-[3,4,5-trihydroxy-6-(hydroxymethyl) oxan-2-YL]-8-(3,4,5-trihydroxyoxan-2-YL) chromen-4-one | C_26_H_28_O_13_ | [M+H]^+^ | 549.1603 | 549.1602 | -0.20 | 531.1404,513.1404,495.1310,483.1303,  477.1189,465.1201,453.1209,387.0874 | Flavonoids |
| 65 | 5.40 | Rubrofusarin gentiobioside | C_27_H_32_O_15_ | [M+H]^+^ | 597.1814 | 597.1812 | -0.32 | 435.1305,273.0788 | Naphthopyrones |
| 66 | 5.43 | Chrysin-5-O-glucoside | C_23_H_24_O_12_ | [M-H]^-^ | 491.1195 | 491.1195 | 0.01 | 329.0677,314.0434,299.0200 | Flavonoids |
| 67 | 5.47 | 3-dihydrocadambine | C_27_H_34_N_2_O_10_ | [M+H]^+^ | 547.2286 | 547.2285 | -0.14 | 385.1770,367.1652,353.1513 | Alkaloids |
| 68 | 5.49 | Kaempferol-7-neohesperidoside | C_27_H_30_O_15_ | [M-H]^-^ | 593.1512 | 593.1513 | 0.26 | 285.0407,269.0466 | Flavonoids |
| 69 | 5.50 | Isorhynchophyllic acid | C_21_H_26_N_2_O_4_ | [M+H]^+^ | 371.1965 | 371.1961 | -1.06 | 353.1849,269.1647,160.0755 | Alkaloids |
| 70 | 5.56 | Isochlorogenic acid B | C_25_H_24_O_12_ | [M-H]^-^ | 515.1195 | 515.1190 | -0.93 | 407.7233,353.0891,191.0562,173.0449 | Phenolic acids |
| 71 | 5.60 | Kaempferol-3-O-rutinoside | C_27_H_30_O_15_ | [M-H]^-^ | 593.1512 | 593.1513 | 0.24 | 285.0413,269.0478 | Flavonoids |
| 72 | 5.68 | Pinoresinol 4-O-glucoside | C_26_H_32_O_11_ | [M-H]^-^ | 519.1872 | 519.1870 | -0.38 | 357.1361,221.0825,137.0607 | Phenylpropanoids |
| 73 | 5.88 | Verbascoside* | C_29_H_36_O_15_ | [M-H]^-^ | 623.1981 | 623.1982 | 0.04 | 461.1675,179.0357,161.0282,135.0451 | Phenylethanoid glycosides |
| 74 | 5.90 | Norrubrofusarin 6-beta-gentiobioside | C_26_H_30_O_15_ | [M+H]^+^ | 583.1658 | 583.1661 | 0.64 | 275.0544 | Naphthopyrones |
| 75 | 6.01 | Beta-Hydroxyacteoside | C_29_H_36_O_16_ | [M+H]^+^ | 641.2076 | 641.2071 | -0.75 | 333.096,318.0729 | Phenylethanoid glycosides |
| 76 | 6.01 | Acanthoside B | C_28_H_36_O_13_ | [M+NH4]^+^ | 598.2494 | 598.2494 | -0.09 | 419.1694,401.1587,383.1480,265.1066,  235.0963,205.0854,173.0592,167.0699 | Phenylpropanoids |
| 77 | 6.05 | 5-Hydroxy-3-(5-hydroxy-2,4-dimethoxyphenyl)-6-methoxy-7-[3,4,5-trihydroxy-6-(hydroxymethyl) oxan-2-yl] oxychromen-4-one | C_24_H_26_O_13_ | [M+H]^+^ | 523.1446 | 523.1444 | -0.42 | 361.0919,346.0683,331.0458 | Flavonoids |
| 78 | 6.23 | Tatarine A | C_17_H_13_NO_3_ | [M+H]^+^ | 280.0968 | 280.0959 | -3.27 | 264.0655,236.0697,150.0295 | Alkaloids |
| 79 | 6.27 | Asaraldehyde | C_10_H_12_O_4_ | [M+H]^+^ | 197.0808 | 197.0808 | -0.16 | 169.0889,154.0636,138.0702,123.0447 | Aromatic aldehydes |
| 80 | 6.28 | Marmesin | C_14_H_14_O_4_ | [M+H]^+^ | 247.0965 | 247.0961 | -1.61 | 229.0870,205.0857,177.0906 | Phenylpropanoids |
| 81 | 6.35 | Scoparone | C_11_H_10_O_4_ | [M+H]^+^ | 207.0652 | 207.0654 | 1.24 | 175.0388,147.0439,119.0490,91.0545 | Phenylpropanoids |
| 82 | 6.35 | Isochlorogenic acid C | C_25_H_24_O_12_ | [M-H]^-^ | 515.1195 | 515.1184 | -2.13 | 353.0879,191.0558,173.0456 | Phenolic acids |
| 83 | 6.35 | 4H-1-Benzopyran-4-one, 2-(2,6-dihydroxyphenyl)-2,3-dihydro-5,7-dihydroxy-, (2S)- | C_15_H_12_O_6_ | [M+H]^+^ | 289.0707 | 289.0707 | 0.14 | 153.0189 | Flavonoids |
| 84 | 6.39 | (3sinapoyl)  fructofuranosyl-  (6-sinapoyl) glucopyranoside* | C_34_H_42_O_19_ | [M-H]^-^ | 753.2248 | 753.2252 | 0.62 | 547.1675,529.1563,223.0612,205.0525 | Oligosaccharides |
| 85 | 6.42 | Chrysophanol tetraglucoside | C_39_H_50_O_24_ | [M-H]^-^ | 901.2619 | 901.2616 | -0.36 | 647.2047,253.0511,179.0559 | Anthraquinones |
| 86 | 6.46 | Cassiaside C2 | C_39_H_52_O_25_ | [M-H]^-^ | 919.2725 | 919.2715 | -1.09 | 623.7058,271.0619 | Naphthopyrones |
| 87 | 6.51 | 1,7-dihydroxy-3-methoxyxanthone | C_14_H_10_O_5_ | [M+H]^+^ | 259.0601 | 259.0602 | 0.55 | 241.0482,213.0541,185.0598 | Xanthones |
| 88 | 6.53 | Viscidulin III | C_17_H_14_O_8_ | [M+H]^+^ | 347.0761 | 347.0759 | -0.77 | 317.0295,289.0348,169.0134,137.0233 | Flavonoids |
| 89 | 6.54 | Arillanin A | C_33_H_40_O_18_ | [M-H]^-^ | 723.2142 | 723.2139 | -0.44 | 547.1672,223.0613,205.0505,175.0409 | Oligosaccharides |
| 90 | 6.58 | Isocorynoxeine | C_22_H_26_N_2_O_4_ | [M+H]^+^ | 383.1965 | 383.1962 | -0.82 | 351.1707,267.1490,160.0764 | Alkaloids |
| 91 | 6.59 | Cassiaside | C_20_H_20_O_10_ | [M-H]^-^ | 419.0984 | 419.0976 | -1.84 | 257.0462,213.0557 | Naphthopyrones |
| 92 | 6.60 | Cassitoroside | C_25_H_32_O_14_ | [M+H]^+^ | 557.1865 | 557.1864 | -0.08 | 263.0933,245.0812 | Anthraquinones |
| 93 | 6.61 | Hispidulin 7-glucuronide | C_22_H_20_O_12_ | [M+H]^+^ | 477.1028 | 477.1028 | 0.12 | 301.0832,286.0535 | Flavonoids |
| 94 | 6.63 | Isorhynchophylline* | C_22_H_28_N_2_O_4_ | [M+H]^+^ | 385.2122 | 385.2117 | -1.27 | 353.1859,269.1647,267.1484,241.1329,  215.1174,187.0867,160.0756,144.0809 | Alkaloids |
| 95 | 6.65 | Matairesinoside | C_26_H_32_O_11_ | [M+H]^+^ | 521.2017 | 521.2015 | -0.50 | 359.1492,341.1371,311.1268,235.0965,  205.0857,189.0928 | Phenylpropanoids |
| 96 | 6.71 | Corynoxeine | C_22_H_26_N_2_O_4_ | [M+H]^+^ | 383.1965 | 383.1962 | -0.82 | 351.1707,267.1490,224.1275,160.0764,  108.0809 | Alkaloids |
| 97 | 6.71 | Scutellarein* | C_15_H_10_O_6_ | [M+H]^+^ | 287.0550 | 287.0548 | -0.77 | 269.0435,169.0130,123.0073 | Flavonoids |
| 98 | 6.78 | Methoxyeugenol | C_11_H_14_O_3_ | [M+H]^+^ | 195.1016 | 195.1011 | -2.20 | 163.0396,154.0627,139.0388,135.1173,  107.0494 | Phenylpropanoids |
| 99 | 6.83 | Cynaroside | C_21_H_20_O_11_ | [M-H]^-^ | 447.0933 | 447.0931 | -0.51 | 285.0400,284.0342,256.0388 | Flavonoids |
| 100 | 6.98 | Gluco-Aurantioobtusin | C_23_H_24_O_12_ | [M-H]^-^ | 491.1195 | 491.1200 | 0.99 | 476.1010,461.0737,433.0784,329.0680,  313.0380,299.0222,285.0410,270.0174,  242.0224 | Anthraquinones |
| 101 | 6.99 | Cassiaside C | C_27_H_32_O_15_ | [M+H]^+^ | 597.1814 | 597.1815 | 0.16 | 435.1290,417.1199,399.1083,339.0870,  351.0861,273.0875 | Naphthopyrones |
| 102 | 7.05 | Rhoifolin | C_27_H_30_O_14_ | [M-H]^-^ | 577.1563 | 577.1558 | -0.82 | 253.0511 | Flavonoids |
| 103 | 7.07 | (16R)-Dihydrositsirikine | C_21_H_28_N_2_O_3_ | [M+H]^+^ | 357.2170 | 357.2170 | 0.00 | 266.1748,226.1431,144.0806 | Alkaloids |
| 104 | 7.10 | Jionoside D | C_30_H_38_O_15_ | [M-H]^-^ | 637.2138 | 637.2136 | -0.28 | 491.1569,475.1854,461.1669,315.1091,  307.1319,193.0498,175.0407,161.0245 | Phenylethanoid glycosides |
| 105 | 7.12 | Baicalein-7-o-glucoside | C_21_H_20_O_10_ | [M+H]^+^ | 433.1129 | 433.1124 | -1.11 | 271.0683 | Flavonoids |
| 106 | 7.13 | Tenuifoliside A | C_31_H_38_O_17_ | [M-H]^-^ | 681.2036 | 681.2040 | 0.56 | 443.1196,137.0253 | Oligosaccharides |
| 107 | 7.23 | Baicalin* | C_21_H_18_O_11_ | [M-H]^-^ | 445.0776 | 445.0780 | 0.82 | 269.0551,251.0361,241.0518,223.0416,  175.0276,169.0667,153.0715 | Flavonoids |
| 108 | 7.25 | Feruloyltyramine | C_18_H_19_NO_4_ | [M+H]^+^ | 314.1387 | 314.1386 | -0.11 | 177.0545,145.0283,117.0335 | Alkaloids |
| 109 | 7.29 | Rhynchophylline* | C_22_H_28_N_2_O_4_ | [M+H]^+^ | 385.2122 | 385.2122 | -0.07 | 353.1866,269.1657,267.1491,241.1335,  215.1179,160.0780,144.0806 | Alkaloids |
| 110 | 7.33 | 3,4,5-Trihydroxy-6-[5-hydroxy-2-(4-hydroxy-3-methoxyphenyl)-3-methoxy-4-oxochromen-7-yl] oxyoxane-2-carboxylic acid | C_23_H_22_O_13_ | [M-H]^-^ | 505.0988 | 505.0971 | -3.37 | 329.0668,314.0431,299.0200 | Flavonoids |
| 111 | 7.39 | 5,7,2'-Trihydroxy-6-methoxyflavone 7-O-glucoside | C_22_H_22_O_11_ | [M-H]^-^ | 461.1089 | 461.1072 | -3.77 | 299.0562,285.0407 | Flavonoids |
| 112 | 7.43 | 3,4,5-Trimethoxycinnamic acid | C_12_H_14_O_5_ | [M+H]^+^ | 239.0914 | 239.0912 | -0.64 | 206.0574,193.0864,191.0353,178.0624,  176.0463,163.0396 | Organic acids |
| 113 | 7.52 | 5,2',6'-Trihydroxy-7,8-dimethoxyflavone 2'-glucuronide | C_23_H_22_O_13_ | [M-H]^-^ | 505.0988 | 505.0978 | -1.85 | 329.0679,314.0435,299.0202 | Flavonoids |
| 114 | 7.66 | Quercetin* | C_15_H_10_O_7_ | [M+H]^+^ | 303.0499 | 303.0497 | -0.77 | 285.0378,257.0451,229.0493,201.0544,  153.0175,137.0228 | Flavonoids |
| 115 | 7.67 | Corynoxine | C_22_H_28_N_2_O_4_ | [M+H]^+^ | 385.2122 | 385.2119 | -0.70 | 353.1852,269.1644,241.1331,160.0751 | Alkaloids |
| 116 | 7.77 | Phillygenin | C_21_H_24_O_6_ | [M+H]^+^ | 373.1646 | 373.1644 | -0.52 | 355.1550,337.1467,305.1174,269.0803,  241.0857 | Phenylpropanoids |
| 117 | 7.79 | Phillyrin | C_27_H_34_O_11_ | [M+HCOO]^-^ | 579.2072 | 579.2087 | 2.61 | 371.1521,356.1273 | Phenylpropanoids |
| 118 | 7.82 | Isomartynoside | C_31_H_40_O_15_ | [M-H]^-^ | 651.2294 | 651.2280 | -2.19 | 421.5084,193.0503,175.0407 | Phenylethanoid glycosides |
| 119 | 7.87 | Cassiaside B | C_26_H_30_O_14_ | [M-H]^-^ | 565.1563 | 565.1552 | -1.88 | 271.062 | Naphthopyrones |
| 120 | 7.88 | Vallesiachotamine | C_21_H_22_N_2_O_3_ | [M+H]^+^ | 351.1703 | 351.1708 | 1.39 | 333.1762,319.1440,249.1384 | Alkaloids |
| 121 | 8.15 | Glychionide A | C_21_H_18_O_11_ | [M-H]^-^ | 445.0776 | 445.0776 | -0.12 | 269.0563,241.0519,225.0566,197.0613,  175.0254,171.0451 | Flavonoids |
| 122 | 8.18 | Chrysin 8-C-glucoside | C_21_H_20_O_9_ | [M+H]^+^ | 417.1180 | 417.1174 | -1.47 | 255.0653 | Flavonoids |
| 123 | 8.25 | Tenuifoliside C | C_35_H_44_O_19_ | [M-H]^-^ | 767.2404 | 767.2398 | -0.80 | 529.1556,265.0721,223.0617,205.0507 | Oligosaccharides |
| 124 | 8.30 | Viscidulin II | C_17_H_14_O_7_ | [M+H]^+^ | 331.0812 | 331.0809 | -0.94 | 316.0576,298.0463,270.0519,242.0573,  197.0443 | Flavonoids |
| 125 | 8.30 | Wogonin 5-glucoside | C_22_H_22_O_10_ | [M+H]^+^ | 447.1286 | 447.1270 | -3.50 | 285.0815,271.0605,270.0544 | Flavonoids |
| 126 | 8.31 | Alizarin | C_14_H_8_O_4_ | [M+H]^+^ | 241.0495 | 241.0493 | -1.20 | 213.0553,185.0598,157.0650,129.0697 | Anthraquinones |
| 127 | 8.32 | Martynoside | C_31_H_40_O_15_ | [M-H]^-^ | 651.2294 | 651.2287 | -1.09 | 193.0507,175.0402 | Phenylethanoid glycosides |
| 128 | 8.36 | Chrysin-7-O-glucuronide | C_21_H_18_O_10_ | [M-H]^-^ | 429.0827 | 429.0828 | 0.17 | 253.0613,175.0265,113.0275,99.0091,  85.0309 | Flavonoids |
| 129 | 8.39 | 1-Hydroxy-3,7-dimethoxyxanthone | C_15_H_12_O_5_ | [M+H]^+^ | 273.0758 | 273.0756 | -0.54 | 255.0647,227.0697 | Xanthones |
| 130 | 8.39 | Prunin | C_21_H_22_O_10_ | [M+H]^+^ | 435.1286 | 435.1284 | -0.44 | 273.0832 | Flavonoids |
| 131 | 8.43 | Oroxylin A glucoronide | C_22_H_20_O_11_ | [M+H]^+^ | 461.1078 | 461.1079 | 0.23 | 285.0786,270.0532 | Flavonoids |
| 132 | 8.48 | Luteolin | C_15_H_10_O_6_ | [M+H]^+^ | 287.0550 | 287.0542 | -2.81 | 272.0560,153.0178,135.0435 | Flavonoids |
| 133 | 8.53 | DiosMetin 7-O-beta-D-glucuronide | C_22_H_20_O_12_ | [M+H]^+^ | 477.1028 | 477.1027 | -0.06 | 301.0824,286.0552 | Flavonoids |
| 134 | 8.65 | Cucumin C | C_15_H_16_O_3_ | [M+H]^+^ | 245.1172 | 245.1172 | -0.06 | 229.0861,199.1011,181.1007 | Terpenoids |
| 135 | 8.79 | Glucoemodin | C_21_H_20_O_10_ | [M-H]^-^ | 431.0984 | 431.0975 | -2.04 | 299.4165,269.0463,255.0662 | Anthraquinones |
| 136 | 8.83 | Chryso-obtusin glucoside | C_25_H_28_O_12_ | [M+H]^+^ | 521.1654 | 521.1648 | -0.99 | 359.1238,329.0665 | Anthraquinones |
| 137 | 8.85 | Oroxindin* | C_22_H_20_O_11_ | [M-H]^-^ | 459.0933 | 459.0932 | -0.23 | 283.0625,268.0389,175.0255 | Flavonoids |
| 138 | 8.99 | Dihydrooroxylin A | C_16_H_14_O_5_ | [M+H]^+^ | 287.0914 | 287.0909 | -1.63 | 183.0298,168.0053,140.0106,131.0492 | Flavonoids |
| 139 | 9.01 | Tectoridin | C_22_H_22_O_11_ | [M-H]^-^ | 461.1089 | 461.1077 | -2.61 | 285.0786,175.0249,165.9911,137.9961 | Flavonoids |
| 140 | 9.02 | Yohimbine | C_21_H_26_N_2_O_3_ | [M+H]^+^ | 355.2016 | 355.2014 | -0.71 | 224.1294,212.1283,170.0965,144.0816 | Alkaloids |
| 141 | 9.04 | Physcion-8-O-β-D-glucopyranoside | C_22_H_22_O_10_ | [M-H]^-^ | 445.1140 | 445.1128 | -2.66 | 283.0611,269.0459 | Anthraquinones |
| 142 | 9.07 | 5,2',6'-Trihydroxy-6,7,8-trimethoxyflavone | C_18_H_16_O_8_ | [M+H]^+^ | 361.0918 | 361.0917 | -0.32 | 346.0686,331.0457,313.0348,227.0547,  197.0077,169.0127 | Flavonoids |
| 143 | 9.11 | Hispidulin | C_16_H_12_O_6_ | [M-H]^-^ | 299.0561 | 299.0554 | -2.22 | 284.0330,136.9884 | Flavonoids |
| 144 | 9.16 | Geissoschizine methyl ether | C_22_H_26_N_2_O_3_ | [M+H]^+^ | 367.2016 | 367.2017 | 0.16 | 335.1758,251.1536,236.1292,224.1290,  192.1018,170.0966,144.0851,130.0655 | Alkaloids |
| 145 | 9.21 | Kaempferol | C_15_H_10_O_6_ | [M+H]^+^ | 287.0550 | 287.0545 | -1.84 | 153.0178 | Flavonoids |
| 146 | 9.42 | Norwogonin | C_15_H_10_O_5_ | [M+H]^+^ | 271.0601 | 271.0604 | 1.07 | 253.0494,225.0552,197.0598,169.0148,  141.0701,123.0083 | Flavonoids |
| 147 | 9.58 | Polygalasaponin Xxviii | C_53_H_84_O_24_ | [M-H]^-^ | 1103.5280 | 1103.5276 | -0.31 | 1073.5182,455.3180,425.3075 | Terpenoids |
| 148 | 9.65 | Hirsuteine | C_22_H_26_N_2_O_3_ | [M+H]^+^ | 367.2016 | 367.2019 | 0.65 | 335.1773,298.1452,251.1550,236.1286,  224.1292,210.1127,192.1021,182.0964,  170.0985,144.0906 | Alkaloids |
| 149 | 9.68 | Alpinetin | C_16_H_14_O_4_ | [M+H]^+^ | 271.0965 | 271.0959 | -2.12 | 167.0338,152.0101,124.0150 | Flavonoids |
| 150 | 9.76 | (9E)-11a-hydroxy-3,6,10-trimethyl-6,7,8,11-tetrahydro-4H-cyclodeca[b]furan-2,5-dione | C_15_H_20_O_4_ | [M+H]^+^ | 265.1434 | 265.1436 | 0.63 | 247.1334,229.1228,219.1386,201.1273,  173.1313,161.0958 | Terpenoids |
| 151 | 9.80 | Matairesinol | C_20_H_22_O_6_ | [M+H]^+^ | 359.1489 | 359.1489 | -0.04 | 235.0962,205.0853,151.0396,123.0435 | Phenylpropanoids |
| 152 | 10.02 | Baicalein | C_15_H_10_O_5_ | [M+H]^+^ | 271.0601 | 271.0602 | 0.53 | 253.0494,225.0546,169.0132,123.0078 | Flavonoids |
| 153 | 10.10 | 1-Desmethylobtusin | C_17_H_14_O_7_ | [M+H]^+^ | 331.0812 | 331.0814 | 0.50 | 316.0599,301.0415,273.0388 | Anthraquinones |
| 154 | 10.14 | Strictosamide | C_26_H_30_N_2_O_8_ | [M+H]^+^ | 499.2075 | 499.2073 | -0.29 | 337.1597,267.1137,171.0940,144.0813 | Alkaloids |
| 155 | 10.15 | Hirsutine | C_22_H_28_N_2_O_3_ | [M+H]^+^ | 369.2173 | 369.2170 | -0.65 | 337.1911,226.1455,170.0974,144.0901 | Alkaloids |
| 156 | 10.21 | Ncgc00385982-01_C27H30N2O10_ | C_27_H_30_N_2_O_10_ | [M+H]^+^ | 543.1973 | 543.1973 | -0.01 | 381.1439,363.1344,335.1386,311.1031,  265.0975,188.0702 | Alkaloids |
| 157 | 10.29 | Scutellarein 4'-methyl ether | C_16_H_12_O_6_ | [M+H]^+^ | 301.0707 | 301.0706 | -0.22 | 286.0509,168.0061,140.0104 | Flavonoids |
| 158 | 10.32 | Tectorigenin | C_16_H_12_O_6_ | [M-H]^-^ | 299.0561 | 299.0557 | -1.34 | 284.0351,267.0299,165.9908,137.9957 | Flavonoids |
| 159 | 10.61 | Curcolone | C_15_H_18_O_3_ | [M+H]^+^ | 247.1329 | 247.1327 | -0.49 | 229.1234,211.1120,201.1274,183.1168,  143.0856,129.0697 | Terpenoids |
| 160 | 10.64 | Tectochrysin | C_16_H_12_O_4_ | [M+H]^+^ | 269.0808 | 269.0804 | -1.51 | 253.0493,237.0540,225.0539,197.0599,  181.0634,167.1026 | Flavonoids |
| 161 | 10.67 | Curdionolide B | C_15_H_20_O_3_ | [M+H]^+^ | 249.1485 | 249.1484 | -0.51 | 231.1375,213.1277,203.1435,185.1334,  161.1203,147.0806 | Terpenoids |
| 162 | 10.81 | 1,7-Dihydroxy-2,3-dimethoxyxanthone | C_15_H_12_O_6_ | [M+H]^+^ | 289.0707 | 289.0705 | -0.51 | 274.0471,256.0363,228.0421,200.0466 | Xanthones |
| 163 | 10.88 | Aurantio-obtusin | C_17_H_14_O_7_ | [M+H]^+^ | 331.0812 | 331.0813 | 0.25 | 316.0627,298.0569,270.0580,242.0633 | Anthraquinones |
| 164 | 10.89 | Tenuifolin | C_36_H_56_O_12_ | [M-H]^-^ | 679.3699 | 679.3696 | -0.45 | 455.3181,425.3078 | Terpenoids |
| 165 | 11.18 | 1,2,3,6,7-Pentamethoxyxanthone | C_18_H_18_O_7_ | [M+H]^+^ | 347.1125 | 347.1125 | 0.03 | 331.0816,317.0660,303.0861,289.0705 | Xanthones |
| 166 | 11.22 | 5,7,3'-Trihydroxy-3,6,4',5'-tetramethoxyflavone | C_19_H_18_O_9_ | [M+H]^+^ | 391.1024 | 391.1025 | 0.29 | 376.0780,361.0551,343.0443,333.0607,  197.0071 | Flavonoids |
| 167 | 11.23 | 2-Hydroxyemodin | C_15_H_10_O_6_ | [M+H]^+^ | 287.0550 | 287.0550 | -0.10 | 259.0594,245.0443,231.0648,217.0489 | Anthraquinones |
| 168 | 11.24 | Isorhamnetin | C_16_H_12_O_7_ | [M+H]^+^ | 317.0656 | 317.0657 | 0.32 | 302.0433,285.0397,274.0485,257.0434,  229.0490,153.0182 | Flavonoids |
| 169 | 11.49 | Negletein | C_16_H_12_O_5_ | [M+H]^+^ | 285.0758 | 285.0754 | -1.25 | 270.0520,168.0046,140.0112 | Flavonoids |
| 170 | 11.60 | Quercetin pentamethyl ether | C_20_H_20_O_7_ | [M+H]^+^ | 373.1282 | 373.1278 | -0.97 | 343.0812,329.1026,165.0540,151.0391 | Flavonoids |
| 171 | 11.81 | Curdione | C_15_H_24_O_2_ | [M+H]^+^ | 237.1849 | 237.1845 | -1.56 | 219.1736,201.1632,175.1473,159.1168,  145.1009 | Terpenoids |
| 172 | 11.91 | 6-Demethoxytangeretin | C_19_H_18_O_6_ | [M+H]^+^ | 343.1176 | 343.1170 | -1.80 | 313.0712,285.0756,211.0776,181.0131,  153.0183 | Flavonoids |
| 173 | 12.01 | Saikosaponin C | C_48_H_78_O_17_ | [M+H]^+^ | 927.5312 | 927.5316 | 0.42 | 909.4987,781.4624,439.3367,421.3466,  403.3368,309.1169 | Terpenoids |
| 174 | 12.08 | Tenuifoliside E | C_41_H_48_O_22_ | [M-H]^-^ | 891.2565 | 891.2543 | -2.46 | 667.0767,633.4437,613.1726,387.8969 | Oligosaccharides |
| 175 | 12.11 | Wogonin | C_16_H_12_O_5_ | [M+H]^+^ | 285.0758 | 285.0757 | -0.17 | 270.0544,252.0423,242.0578,179.0498,  151.0545 | Flavonoids |
| 176 | 12.14 | Emodinanthrone | C_15_H_12_O_4_ | [M+H]^+^ | 257.0808 | 257.0811 | 1.19 | 241.0492,227.0344,213.0543,199.0393,  171.0428 | Anthraquinones |
| 177 | 12.30 | Aerugidiol | C_15_H_22_O_3_ | [M+H]^+^ | 251.1642 | 251.1640 | -0.71 | 233.1541,215.1428,205.1589,191.1423,  173.1320,149.0956 | Terpenoids |
| 178 | 12.30 | Chrysin | C_15_H_10_O_4_ | [M+H]^+^ | 255.0652 | 255.0653 | 0.37 | 153.0186 | Flavonoids |
| 179 | 12.38 | Skullcapflavone I | C_17_H_14_O_6_ | [M+H]^+^ | 315.0863 | 315.0861 | -0.68 | 300.0678,285.0459,282.0567,257.0477,  197.0602,182.9975 | Flavonoids |
| 180 | 12.43 | Alpha-curcumene | C_15_H_22_ | [M+H]^+^ | 203.1794 | 203.1793 | -0.74 | 175.1476,161.1321,147.1163,133.1007,  119.0857,105.0698 | Terpenoids |
| 181 | 12.55 | Pinocembrin | C_15_H_12_O_4_ | [M+H]^+^ | 257.0808 | 257.0802 | -2.32 | 153.0178,131.0491,103.0547 | Flavonoids |
| 182 | 12.62 | Chrysoobtusin | C_19_H_18_O_7_ | [M+H]^+^ | 359.1125 | 359.1125 | -0.15 | 329.0663,311.0553,283.0603,255.0659 | Anthraquinones |
| 183 | 12.69 | Skullcapflavone II | C_19_H_18_O_8_ | [M+H]^+^ | 375.1074 | 375.1077 | 0.67 | 360.063,345.0669,327.0540,227.0564,  197.0126 | Flavonoids |
| 184 | 12.73 | Isozedoarondiol | C_15_H_24_O_3_ | [M+H]^+^ | 253.1798 | 253.1785 | -5.27 | 235.1681,217.1581,189.1637,175.1476,  147.1164 | Terpenoids |
| 185 | 12.73 | Oroxylin A | C_16_H_12_O_5_ | [M+H]^+^ | 285.0758 | 285.0759 | 0.52 | 270.0521,168.0052 | Flavonoids |
| 186 | 12.80 | Tetramethylscutellarein | C_19_H_18_O_6_ | [M+H]^+^ | 343.1176 | 343.1175 | -0.25 | 327.0854,313.0701,299.0913,282.0982,  153.0176 | Flavonoids |
| 187 | 12.91 | 1,2,3,7-Tetramethoxyxanthone | C_17_H_16_O_6_ | [M+H]^+^ | 317.1020 | 317.1019 | -0.30 | 301.0717,287.0549,273.0750,259.0606 | Xanthones |
| 188 | 12.92 | Beta-Asarone | C_12_H_16_O_3_ | [M+H]^+^ | 209.1172 | 209.1172 | 0.00 | 194.0946,181.0863,179.0705,163.0754,  151.0755,136.0520,121.0648,91.0543 | Phenylpropanoids |
| 189 | 13.08 | Zedoarondiol | C_15_H_24_O_3_ | [M+H]^+^ | 253.1798 | 253.1797 | -0.42 | 235.1685,217.1584,189.1630,175.1470,  147.1163 | Terpenoids |
| 190 | 13.09 | Procurcumenol | C_15_H_22_O_2_ | [M+H]^+^ | 235.1693 | 235.1693 | 0.22 | 217.1583,189.1631,175.1147,161.0985,  133.1011,119.0854 | Terpenoids |
| 191 | 13.26 | Methyl eugenol | C_11_H_14_O_2_ | [M+H]^+^ | 179.1067 | 179.1067 | 0.40 | 151.0786,121.0649,107.0496,91.0548 | Phenylpropanoids |
| 192 | 13.34 | Hibiscetin heptamethyl ether | C_22_H_24_O_9_ | [M+H]^+^ | 433.1493 | 433.1485 | -1.90 | 418.1249,403.1023,385.0920 | Flavonoids |
| 193 | 13.41 | Saikosaponin A* | C_42_H_68_O_13_ | [M-H]^-^ | 779.4587 | 779.4573 | -1.84 | 617.4052 | Terpenoids |
| 194 | 13.47 | Obtusin | C_18_H_16_O_7_ | [M-H]^-^ | 343.0823 | 343.0823 | -0.02 | 328.0653,313.0392,285.0419 | Anthraquinones |
| 195 | 13.61 | Alpha-Asarone | C_12_H_16_O_3_ | [M+H]^+^ | 209.1172 | 209.1173 | 0.55 | 181.0861,179.0705,151.0752,135.0804,  121.0648,91.0542 | Phenylpropanoids |
| 196 | 13.67 | Obtusifolin | C_16_H_12_O_5_ | [M+H]^+^ | 285.0758 | 285.0756 | -0.56 | 270.0589,253.0496,242.0594,225.0554,  211.0764 | Anthraquinones |
| 197 | 13.69 | Oxyphyllacinol | C_20_H_26_O_3_ | [M+H]^+^ | 315.1955 | 315.1950 | -1.37 | 297.1846,285.0406,227.1796,145.1005,  131.0855 | Diarylheptanoids |
| 198 | 13.72 | 1,2-Dihydrocurcumin | C_21_H_22_O_6_ | [M+H]^+^ | 371.1489 | 371.1481 | -2.20 | 355.0680,335.1294,151.0756,135.0440 | Diarylheptanoids |
| 199 | 13.93 | Saikosaponin B1 | C_42_H_68_O_13_ | [M-H]^-^ | 779.4587 | 779.4580 | -0.91 | 617.4081 | Terpenoids |
| 200 | 14.19 | Saikosaponin B2 | C_42_H_68_O_13_ | [M-H]^-^ | 779.4587 | 779.4579 | -1.10 | 617.4069 | Terpenoids |
| 201 | 14.99 | 2-(3-Methoxy-4-hydroxyphenyl)-5-(3,4-dimethoxyphenyl)-3,4-dimethyltetrahydrofuran | C_21_H_26_O_5_ | [M+H]^+^ | 359.1853 | 359.1850 | -0.96 | 235.1333,221.1171,203.1063,179.1069,  151.0756,137.0579 | Phenylpropanoids |
| 202 | 15.00 | Oxyphyllanene C | C_14_H_18_O_3_ | [M+H]^+^ | 235.1329 | 235.1329 | 0.00 | 217.1225,202.0989,151.0754,133.1013 | Terpenoids |
| 203 | 15.02 | Emodin* | C_15_H_10_O_5_ | [M+H]^+^ | 271.0601 | 271.0601 | 0.15 | 229.0493,225.0545,201.0550,197.0605,  173.0596,169.0657,145.0645,141.0695 | Anthraquinones |
| 204 | 15.09 | 6''-O-acetyl-saikosaponin B2 | C_44_H_70_O_14_ | [M-H]^-^ | 821.4693 | 821.4680 | -1.56 | 779.4591,617.4035 | Terpenoids |
| 205 | 15.30 | Cucumin A | C_15_H_16_O_2_ | [M+H]^+^ | 229.1223 | 229.1224 | 0.29 | 213.0960,201.1271,185.0953,167.0857,  153.0698 | Terpenoids |
| 206 | 15.85 | Senkyunolide H | C_12_H_16_O_4_ | [M+H]^+^ | 225.1121 | 225.1116 | -2.32 | 210.0877,193.0854,177.0554,167.0701,  165.0908,153.0550 | Phthaleins |
| 207 | 15.87 | Toralactone | C_15_H_12_O_5_ | [M+H]^+^ | 273.0758 | 273.0756 | -0.41 | 258.0518,230.0567,212.0463,184.0507 | Naphthopyrones |
| 208 | 15.91 | 2''-O-Acetylsaikosaponin A | C_44_H_70_O_14_ | [M-H]^-^ | 821.4693 | 821.4667 | -3.10 | 779.4590,617.4036 | Terpenoids |
| 209 | 16.24 | Senkyunolide I | C_12_H_16_O_4_ | [M+H]^+^ | 225.1121 | 225.1114 | -3.24 | 210.0876,193.0858,177.0542,167.0703,  165.0911,153.0548 | Phthaleins |
| 210 | 16.31 | Oxyphyllanene B | C_12_H_14_O_2_ | [M+H]^+^ | 191.1067 | 191.1067 | -0.05 | 173.0958,163.1120,149.0595,145.1016,  131.0490,117.0701 | Terpenoids |
| 211 | 16.72 | Saucernetin | C_22_H_28_O_5_ | [M+H]^+^ | 373.2010 | 373.2009 | -0.15 | 235.1335,217.1225,202.0981,179.1069,  165.0550,151.0754 | Phenylpropanoids |
| 212 | 16.88 | Oxyphyllanene A | C_12_H_16_O_2_ | [M+H]^+^ | 193.1223 | 193.1219 | -2.37 | 178.0547,165.0908,133.0644,119.0490 | Terpenoids |
| 213 | 17.00 | (+)-Grandisin | C_24_H_32_O_7_ | [M+H]^+^ | 433.2221 | 433.2220 | -0.20 | 265.1464,247.1428,232.1105,216.1222,  209.1168,195.1009,181.0893 | Phenylpropanoids |
| 214 | 17.01 | Zedoarofuran | C_15_H_20_O_4_ | [M+H]^+^ | 265.1434 | 265.1433 | -0.39 | 247.1332,232.1101,216.1145,201.0908,  173.0958 | Terpenoids |
| 215 | 17.01 | Zederone | C_15_H_18_O_3_ | [M+H]^+^ | 247.1329 | 247.1329 | 0.01 | 217.0858,201.0910,173.0959 | Terpenoids |
| 216 | 17.35 | Germacrone | C_15_H_22_O | [M+H]^+^ | 219.1743 | 219.1742 | -0.50 | 204.1509,189.1270,161.1320,149.0961,  135.0802 | Terpenoids |
| 217 | 17.78 | Isourecumenol | C_15_H_22_O_2_ | [M+H]^+^ | 235.1693 | 235.1693 | 0.28 | 217.1589,199.1486,189.1648,175.1119,  161.0958,147.1167,133.1012 | Terpenoids |
| 218 | 17.88 | Chrysophanol* | C_15_H_10_O_4_ | [M+H]^+^ | 255.0652 | 255.0654 | 0.77 | 237.0540,227.0702,209.0596,181.0650,  153.0700 | Anthraquinones |
| 219 | 17.99 | Curcumenol | C_15_H_22_O_2_ | [M+H]^+^ | 235.1693 | 235.1692 | -0.11 | 217.1587,199.1485,189.1639,175.1122,  161.0966 | Terpenoids |
| 220 | 18.94 | Beta-Caryophyllene oxide | C_15_H_24_O | [M+H]^+^ | 221.1900 | 221.1898 | -0.69 | 203.1797,175.1480,161.1321,147.1167,  133.1010,119.0853 | Terpenoids |
| 221 | 19.04 | Senkyunolide G | C_12_H_16_O_3_ | [M+H]^+^ | 209.1172 | 209.1171 | -0.45 | 194.0933,181.0854,178.0981,166.0635,  163.0758,147.0437 | Phthaleins |
| 222 | 19.11 | Alpha-Calacorene | C_15_H_20_ | [M+H]^+^ | 201.1638 | 201.1639 | 0.57 | 159.1171,145.1013,131.0856,117.0698,  105.0700,91.0542 | Terpenoids |
| 223 | 19.13 | Carvacrol | C_10_H_14_O | [M+H]^+^ | 151.1117 | 151.1116 | -1.16 | 133.1013,123.1158,109.0651,105.0697,  91.0542 | Terpenoids |
| 224 | 19.14 | Nootkatone | C_15_H_22_O | [M+H]^+^ | 219.1743 | 219.1744 | 0.34 | 201.1649,173.1320,159.1181,145.1022,  131.0856 | Terpenoids |
| 225 | 19.40 | Corosolic acid | C_30_H_48_O_4_ | [M+H]^+^ | 473.3625 | 473.3626 | 0.09 | 427.3571,247.2058,163.1447 | Terpenoids |
| 226 | 19.42 | Physcione | C_16_H_12_O_5_ | [M+H]^+^ | 285.0758 | 285.0756 | -0.52 | 270.0562,252.0410,242.0578,239.0697,  224.0473,211.0762,196.0515,168.0568 | Anthraquinones |
| 227 | 19.86 | 4,7-Dihydroxy-3-butylphthalide | C_12_H_14_O_4_ | [M+H]^+^ | 223.0965 | 223.0960 | -2.37 | 207.0318,191.0008,149.0233,133.0130 | Phthaleins |
| 228 | 19.89 | Valencen | C_15_H_24_ | [M+H]^+^ | 205.1951 | 205.1952 | 0.49 | 149.1323,135.1168,121.1008,107.0853,  93.0698 | Terpenoids |
| 229 | 20.03 | Aristolone | C_15_H_22_O | [M+H]^+^ | 219.1743 | 219.1744 | 0.06 | 201.1641,163.1121,159.1168,149.0965,  145.1013,131.0856 | Terpenoids |
| 230 | 20.09 | Ligustilide | C_12_H_14_O_2_ | [M+H]^+^ | 191.1067 | 191.1065 | -0.74 | 173.0961,145.1012,117.0699,103.0539 | Phthaleins |
| 231 | 20.10 | Shyobunone | C_15_H_24_O | [M+H]^+^ | 221.1900 | 221.1896 | -1.63 | 203.1795,175.1483,161.1322,147.1173,  133.1014,119.0856,105.0689,91.0542 | Terpenoids |
| 232 | 20.23 | Angelicide | C_24_H_28_O_4_ | [M+H]^+^ | 381.2060 | 381.2061 | 0.15 | 191.1078,173.0957,145.1007,117.0699,  91.0539 | Phthaleins |
| 233 | 20.27 | Alpha-Terpinene | C_10_H_16_ | [M+H]^+^ | 137.1325 | 137.1324 | -0.27 | 122.0760,109.0665,95.0859,81.0693 | Terpenoids |
| 234 | 20.29 | Jaeschkeanadiol | C_15_H_26_O_2_ | [M+H-H_2_O]^+^ | 221.1900 | 221.1902 | 0.90 | 203.1828,175.1495,161.1329,147.1176,  133.1037,119.0866,105.0708,91.0547,  81.0725 | Terpenoids |
| 235 | 20.29 | Calamenene | C_15_H_22_ | [M+H]^+^ | 203.1794 | 203.1795 | 0.54 | 175.1485,161.1328,147.1169,133.1014,  119.0856 | Terpenoids |
| 236 | 20.38 | Eremophilene | C_15_H_24_ | [M+H]^+^ | 205.1951 | 205.1952 | 0.68 | 149.1322,135.1162,121.1010,107.0854,  93.0698 | Terpenoids |
| 237 | 20.44 | 3-Butylphthalide | C_12_H_14_O_2_ | [M+H]^+^ | 191.1067 | 191.1066 | -0.41 | 173.0956,163.1117,149.0597,145.1006,  117.0694,103.0544 | Phthaleins |
| 238 | 20.55 | Levistilide A | C_24_H_28_O_4_ | [M+H]^+^ | 381.2060 | 381.2061 | 0.25 | 191.1120,173.0960,149.0597,135.0441 | Phthaleins |
| 239 | 22.56 | Ursonic acid | C_30_H_46_O_3_ | [M+H]^+^ | 455.3520 | 455.3516 | -0.77 | 437.3443,409.3494,391.3203,201.1642,  189.1637 | Terpenoids |
| 240 | 23.49 | Beta-Caryophyllene | C_15_H_24_ | [M+H]^+^ | 205.1951 | 205.1949 | -0.85 | 149.0235,135.1167,121.1004,107.0847,  93.0687 | Terpenoids |
| 241 | 23.52 | Ursolic Acid | C_30_H_48_O_3_ | [M+H]^+^ | 457.3676 | 457.3677 | 0.11 | 439.3579,411.3607,393.3513,329.1233,  315.2676,249.1352,231.2104,217.1945,  203.1791,191.1795,189.1638,177.1632,  163.1477 | Terpenoids |
| 242 | 27.07 | Lupeol | C_30_H_50_O | [M+H]^+^ | 427.3934 | 427.3932 | -0.67 | 409.3828,191.1793,163.1478,149.1323,  135.1168 | Terpenoids |
| 243 | 27.41 | Oleanolic acid | C_30_H_48_O_3_ | [M+H-H_2_O]^+^ | 439.3571 | 439.3570 | -0.19 | 393.3511,249.1849,217.1950,203.1794,  191.1794,189.1636,175.1475,161.1323 | Terpenoids |

Note: * Confirmed by comparing with the reference standards.

**Table S4** 16 brain-penetrant compounds

| Identification | Sources | Types |
| --- | --- | --- |
| Ferulic acid | Ang | Phenolic acids |
| Zedoalactone C | Cur | Terpenoids |
| Baicalin | Scu | Flavonoids |
| DiosMetin 7-O-beta-D-glucuronide | Scu | Flavonoids |
| Oroxindin | Scu | Flavonoids |
| Baicalein | Scu | Flavonoids |
| Methyl eugenol | Aco | Phenylpropanoids |
| Oroxylin A | Scu | Flavonoids |
| Alpha-Asarone | Aco | Phenylpropanoids |
| 4,7－Dihydroxy－3－butylphthalide | Ang | Phthaleins |
| Eremophilene | Alp | Terpenoids |
| Norwogonin | Scu | Flavonoids |
| Wogonin | Scu | Flavonoids |
| Skullcapflavone I | Scu | Flavonoids |
| Oxyphyllanene B | Alp | Terpenoids |
| Toralactone | Sen | Naphthopyrones |

Ang: *Angelica sinensis* (Oliv.) Diels; Cur: *Curcuma aromatica* Salisb.;

Scu: *Scutellaria baicalensis* Georgi; Aco: *Acorus calamus* var. angustatus Besser;

Alp: *Alpinia oxyphylla* Miq.; Sen: *Senna obtusifolia* (L.) H.S.Irwin & Barneby;
